# The Role of the C-Terminal Lysine of S100P in S100P-Induced Cell Migration and Metastasis

**DOI:** 10.3390/biom11101471

**Published:** 2021-10-06

**Authors:** Thamir M. Ismail, Stephane R. Gross, Tara Lancaster, Philip S. Rudland, Roger Barraclough

**Affiliations:** 1 Institute of Systems, Molecular and Integrative Biology, University of Liverpool, Biosciences Building, Crown Street, Liverpool L69 7ZB, UK; tismail@liverpool.ac.uk (T.M.I.); rudland@liverpool.ac.uk (P.S.R.); 2 College of Health and Life Sciences, Aston University, Aston Triangle, Birmingham B4 7ET, UK; taralancaster94@gmail.com

**Keywords:** S100P, membrane, metastasis, cell migration, myosin llA

## Abstract

S100P protein is a potent inducer of metastasis in a model system, and its presence in cancer cells of patients is strongly associated with their reduced survival times. A well-established Furth Wistar rat metastasis model system, methods for measuring cell migration, and specific inhibitors were used to study pathways of motility-driven metastasis. Cells expressing C-terminal mutant S100P proteins display markedly-reduced S100P-driven metastasis in vivo and cell migration in vitro. These cells fail to display the low focal adhesion numbers observed in cells expressing wild-type S100P, and the mutant S100P proteins exhibit reduced biochemical interaction with non-muscle myosin heavy chain isoform IIA in vitro. Extracellular inhibitors of the S100P-dependent plasminogen activation pathway reduce, but only in part, wild-type S100P-dependent cell migration; they are without effect on S100P-negative cells or cells expressing C-terminal mutant S100P proteins and have no effect on the numbers of focal adhesions. Recombinant wild-type S100P protein, added extracellularly to S100P-negative cells, stimulates cell migration, which is abolished by these inhibitors. The results identify at least two S100P-dependent pathways of migration, one cell surface and the other intracellularly-linked, and identify its C-terminal lysine as a target for inhibiting multiple migration-promoting activities of S100P protein and S100P-driven metastasis.

## 1. Introduction

Elevated levels of some EF-hand-containing, calcium-binding members of the S100 family of proteins are associated with the development of many human cancers [1,2]. Increased immunohistochemical levels of S100P are associated with markedly reduced survival times of patients with breast [3,4], hepatocellular [5], early stage non-small cell lung [6], colon [7,8], and ovarian [9,10] cancers. The associations of S100P with reduced patient survival times are likely to arise from the ability of S100P to induce a metastatic phenotype [3]. However, the molecular pathways by which S100P exerts its metastasis-inducing potential are not yet fully understood [2].

The crystal structure of calcium-bound S100P protein shows that it is a homodimer with each 95-amino-acid monomer displaying the four α-helical structures common to most other S100 proteins [11]. Helix 4 of each monomer ends at amino acid 92, leaving a short, three-amino acid, presumably unstructured sequence, GLK, at the C-terminus [11]. In the apo-form, the presumably unstructured C-terminal region was found to be six amino acid residues longer, KYFEKAGLK, using Nuclear Magnetic Resonance techniques [12]. C-terminal regions of other S100 proteins, for example, S100A4 [13], have been reported to be unstructured and dynamic.

S100 proteins act by interacting with extracellular and intracellular protein targets [14]. Thus, extracellular S100P can interact with the cell-membrane-located RAGE receptor, thereby activating intracellular signalling pathways [15] and with the extracellular plasminogen activator, tPA, to enhance plasmin-dependent cell invasion [16], but its relationship to metastasis is unknown. Intracellular S100P can also interact with cytoskeletal proteins, ezrin [17], IQGAP1 [18], and non-muscle myosin II isoforms A (NMMIIA) and C (NMMIIC) [19], all with high nM range affinities [17,18,19], often causing increased cell migration [19,20]. In the case of NMMIIA, it has been shown using Fluorescence Lifetime Imaging that S100P can interact with NMMIIA in living cells [19]. In cells in which NMMIIA had been knocked down using siRNA, S100P induction lost its effect on cell migration [19]. Furthermore, in an inducible system, expression of S100P led directly to redistribution of NMMIIA filaments and their dissociation. In a cell-free system, S100P can partially dissociate myosin IIA filaments [19]. These observations taken together show that S100P can interact with myosin IIA in vivo and, through dissociation of the NMMIIA filaments, can affect cell migration. It has been shown previously that deletion of the C-terminal lysines of the related metastasis-inducing protein, S100A4, abrogates its metastasis-inducing potential [21,22] in a well-characterised rat model system of human breast cancer metastasis [3,23,24,25]. We now use this well-characterised rat model system of metastasis to show that deletion of the C-terminal lysine of S100P, or its replacement with alanine, reduces the ability of S100P to induce a metastatic phenotype. This approach has identified two separate, coexisting S100P-dependent pathways leading to cell migration, one linked to the cell surface and one intracellular in the same cell system.

## 2. Materials and Methods

### 2.1. Site-Directed Mutagenesis and Recombinant Proteins 

A cDNA encoding the wild-type S100P protein in pET15b vector was subjected to site-directed mutagenesis using a Quikchange™ site-directed mutagenesis kit (Stratagene, La Jolla, CA, USA) with pairs of complementary oligonucleotide primers to generate pET15b vector expressing the two mutant S100Ps, K95A with the C-terminal lysine replaced by alanine and ΔK95 S100P with the C-terminal lysine deleted. The identity of these proteins was confirmed by mass spectrometry. Details of the site directed mutagenesis, production of recombinant protein, and the mass spectrometry are given in Supplementary Methods S1.

### 2.2. Transfection of Mammary Cell Lines

The S100P wild-type and mutated cDNAs in pET-15b were amplified by PCR using a pair of primers bearing (underlined) *Bam*HI and *Hind*III restriction enzyme sites (forward human S100P primer: 5’ CCGGATCC^95^ATGACGGAACTAGAGAC^111^ 3’; reverse-human S100P primer: 5’ GCAAAGCTT^382^TCATTTGAGTCCTGCC^367^ 3’, numbering from GenBank Accession No NM_005980). The PCR products were cloned into pCDNA3.1(-) vector that had been doubly digested with *Bam*HI and *Hind*III. Two to 3-μg recombinant construct were used to transfect the benign rat mammary tumour-derived cell line, Rama 37 [26], using lipofectamine 2000 reagent (InVitrogen, Paisley, UK), and clones and pools of transfected cells were produced, as described previously [16,22], and maintained in medium containing 0.5 mg/mL Geneticin.

### 2.3. Cell Migration Assays

Cell migration assays, using 6.5-mm diameter Transwell permeable devices with 8.0-μm pore size polycarbonate membranes, were carried out, as described previously, using a 1–5% (*v*/*v*) gradient of foetal calf serum and counting random fields [27] or using a 0.5–10% (*v*/*v*) FCS gradient and counting 5 random fields [28]. Scratch migration assays were carried out using a Cell-IQ incubator, as described previously [29] and data analysed as indicated in the figure legends. In some migration experiments, a polyclonal goat S100P antibody (Cat No. AF2957, R&D Systems, Abingdon, UK) was added to the culture medium at the concentration indicated in the Figure legends. This antibody recognises wild type S100P, the K95A, and ΔK95 proteins.

### 2.4. Metastasis Assays In Vivo

Transfected cell clones and pools were subjected to assays for metastasis in rats, as described previously [3,21,23,25]. Transfected cultured Rama 37 cells (2 × 10^6^ cells) syngeneic to Furth Wistar rats (Olac, Banbury, UK) were injected subcutaneously without anaesthesia into the right inguinal mammary gland of 5- to 6-week old virgin females (80–100 g) during the morning in the University of Liverpool’s licensed (PCD 40/2408) Animal Facility, as described previously [23]. Rats were maintained 6 per cage at 19–21 °C, with a minimum 8h of light/day, bedded on straw, and fed Expanded Rat and Mouse Diet No 1 (BP Ltd., Essex, UK) and tap water ad libitum. Tumours were monitored twice weekly and rats euthanised by CO_2_ overdose without anaesthetic after 2 months or earlier if showing signs of stress. After autopsy, the primary and metastasis to the lungs were assessed, blinded and at random, as described previously [21,30]. Power calculations based on a reduction of 50% of metastasis in rats with 90% metastasis for *p* = 0.8, alpha = 0.05, yielded a minimum of 19 rats in each group. Lung tissue for detection of metastases was fixed in formalin, embedded in paraffin wax, sectioned, and stained with haematoxylin and eosin [26]. Lungs were scored positive for metastasis if lung nodules were present or negative if lung nodules were absent.

### 2.5. Immunofluorescence Staining of Cultured Cells 

Rama 37 cells (15,000 cells) expressing wild-type, K95A or ΔK95-mutant S100P, or cells transfected with empty vector were plated onto fibronectin-coated (2.5 μg/cm^2^) glass coverslips in 24-well plates, grown for 48 h, fixed, permeabilized, and blocked [28] (Supplementary Methods S1), before being incubated with primary antibodies against NMMIIA (Covance, now Biolegend, Dedham, MA, USA), non-muscle myosin IIB (NMMIIB, Covance, now Biolegend, Dedham, MA, USA), S100P (BD, Oxford, UK or R&D systems Abingdon, UK), vinculin (Sigma, St Louis, MO, USA) paxillin (Invitrogen, Paisley, UK), or eEF1A (clone CBP-KK1, mouse, Millipore, UK). Secondary antibodies were anti-rabbit or anti-mouse IgG conjugated with FITC (Dako, Ely, UK) or Cy-3 (Stratech Scientific, Norfolk, UK). Quantitation of the immunofluorescence patterns of non-muscle myosin IIA and NMMIIB was carried out, as described previously [19]. For actin staining, 0.6 μM rhodamine phalloidin (Invitrogen, Paisley, UK) was added with secondary antibodies to the other IgGs. Numbers of focal adhesions were counted in randomly-selected cells from 3 independent experiments. For detection of cell surface S100P or eEF1A, the appropriate antibody was added to the culture medium for 1 h prior to fixation and blocking [16].

### 2.6. Isolation of Membrane Fractions 

Cells were scraped into PBS and centrifuged at 300× *g* for 5 min. The pellet was resuspended in homogenisation buffer (250 mM sucrose, 50 mM Tris, 0.25 mM CaCl_2_ pH 7.4), and centrifuged twice at 600× *g* for 5 min. The cell pellet was resuspended in homogenisation buffer, passed through a cell disruption bomb at 4 °C, 800–1000 PSI for 20 min, and the resulting suspension was centrifuged at 550× *g* for 10 min to remove remaining whole cells and nuclei. The supernatant was layered over 35% (*w*/*w*) sucrose/50 mM Tris, pH 7.4, centrifuged at 100,000× *g* for 1 h, the interface collected, diluted with 25 mM sucrose/50 mM Tris pH 7.4, and centrifuged at 100,000× *g* for 30 min. The pellet containing plasma and other membranes was resuspended in 250 mM sucrose/50 mM Tris, pH 7.4.

### 2.7. Interaction between S100P and Recombinant NMMIIA

Binding of recombinant (r)S100P variants to a recombinant protein consisting of the 149 C-terminal amino acids of NMMIIA [31] was tested using a dual-Channel IAsys resonant mirror biosensor (Neosensors, Sedgefield, UK), as described previously [22]. The resulting association and dissociation curves fitted a single-site at least as well as a two-site binding model.

### 2.8. Western Blotting 

Previously described methods of Western blotting of cell extracts from cloned and pooled transfected cells [21,22,32] and membrane fractions [28] were used. 

### 2.9. Statistical Analyses

Fisher’s exact test was used for statistical comparison of tumour incidence and metastasis data in vivo and Mann–Whitney U-test for comparison of numbers of focal adhesions, both using SPSS software. For multiple comparisons within the same data set, Bonferroni or Dunnett multiple comparison with a control ANOVA post-hoc tests were used (Stats Direct Ltd., Cambridge, UK).

## 3. Results

### 3.1. The Effect of C-Terminal Mutants of S100P on Tumorigenesis and Metastasis 

The benign rat mammary tumour-derived cell line, Rama 37, has a long track record of testing for the metastatic potential of experimentally-expressed genes and proteins when injected into the mammary fat pads of immunocompetent syngeneic rats [3,23,25,33,34]. Genes and proteins, including S100P, which confer a metastatic phenotype upon the benign Rama 37 cells in this system, have subsequently been shown to be associated also with reduced patient survival time when they are found in the cancer cells of human patients [3,35,36,37]. Transfected Rama 37 cell clones and pools expressing wild-type S100P, the K95A, or ΔK95 mutant S100P proteins exhibited similar incidences of mammary tumour formation (Table 1). Thus, the C-terminal mutants did not affect the tumorigenicity of the cells. Cells expressing wild-type S100P exhibited high incidences of metastasis, with 70% (cell clone) and 75% (cell pool) of tumour-bearing rats exhibiting lung metastases (Table 1), similar to those reported previously [3]. Examples of immunocytochemical staining of the tumours and metastases arising from injection into the mammary fat pads of syngeneic rats of Rama 37 cells expressing wild-type S100P have been published previously [3]. The cell clone and pool expressing K95A mutant S100P protein exhibited incidences of metastasis of 33% and 47%, which were lower than those of the wild-type clone and pool (Table 1). The transfected cell clones and pools expressing ΔK95 mutant S100P protein exhibited incidences of metastasis (clone 16%, pool 11%), which were lower than wild-type S100P-expressing clone and pool (Table 1). Overall, mutation/removal of the C-terminal amino acid of S100P significantly reduces its ability to cause metastasis in a rodent model system in vivo.

### 3.2. S100P Mutants and Altered Cytoskeletal Organisation 

Wild-type rS100P protein bound to rNMMIIA in vitro with a Kd of 370–470 nM (Supplementary Table S1 and Figure S1), consistent with previously published values of 430–500 nM [19]. The K95A and ΔK95 mutant rS100P proteins exhibited 6 to 13-fold reduced affinity for immobilised rNMMIIA (equilibrium Kd, 2.3 μM and 2.7 μM; kinetic Kd, 4.1 μM and 6 μM, respectively) than the wild-type protein, mainly due to an 8 to 13-fold increased rate of dissociation (Supplementary Table S1). The observation that the association rate of S100P with NMMIIA in vitro was not affected by the C-terminal mutations but that the dissociation rate was suggests that the C-terminal lysine might be required not for initial binding but for structural stability of the complex.

**Figure 1:**
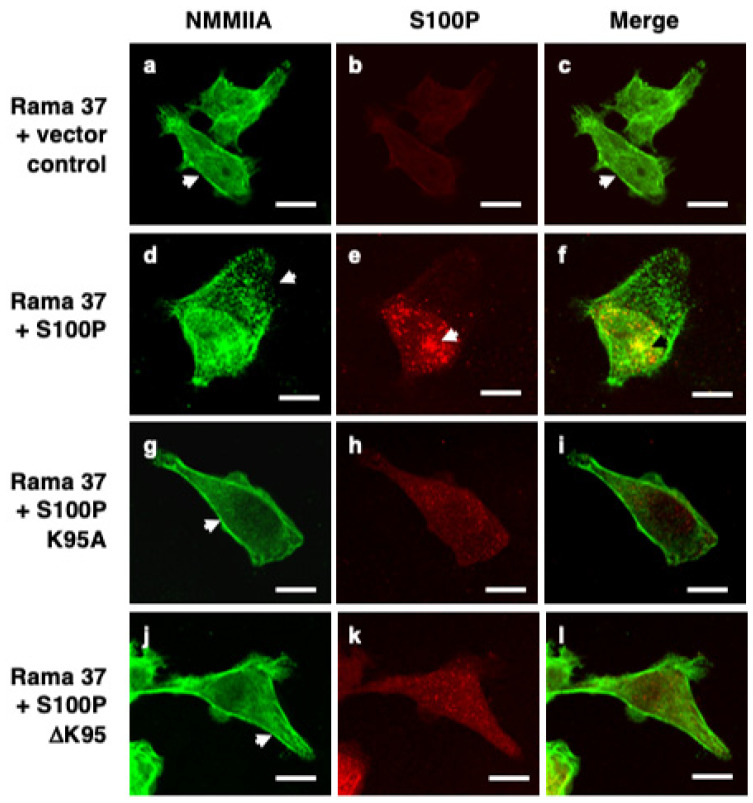
Immunofluorescence localisation of NMMIIA and S100P in cloned transfected cells. Rama 37 cells transfected with empty expression vector (Rama 37 + vector control; (**a**–**c**)) or Rama 37 cells overexpressing wild-type S100P (Rama 37 + S100P; (**d**–**f**)), K95A mutant S100P (Rama 37 + S100P K95A; (**g**–**i**)), or ΔK95 mutant S100P (Rama 37 + S100P ΔK95; (**j**–**l**)) were grown on fibronectin-coated coverslips for 48 h prior to fixation, permeabilisation, and staining using secondary antibody coupled to fluorescein isothiocyanate for NMMIIA (**a**,**d**,**g**,**j**) or Cy-3 for S100P (**b**,**e**,**h**,**k**). Cells were then mounted and viewed using a Zeiss LSM510 confocal laser scanning microscope. Merged images (**c**,**f**,**i**,**l**) are shown with overlaps in yellow. Arrows in (**a**,**c**,**g**,**j**) show filamental structures along the edge of the cell. The arrow in (**d**) shows foci of NMMIIA staining near the leading edge of the cell, which did not co-localise with S100P (**f**). The arrows in (**e**,**f**) show a large focus of S100P co-staining with NMMIIA in the perinuclear region. Bars, 10 μm in all panels.

Immunofluorescence staining of fixed, control, S100P-negative Rama 37 cells revealed cytoplasmic localisation of NMMIIA filamental structures along the edges of the cells (Figure 1a). In cells expressing wild-type S100P, there was a 20.5-fold reduction in the proportion of cells exhibiting this control cell distribution of NMMIIA (Supplementary Table S2; *p* < 0.0001). Whilst there was some background, possibly non-specific, staining for S100P in the S100P-negative cells (Figure 1b and Figure 3B) that was not observed in Western blots (Figure 3A below and Supplementary Material S1 of Clarke et al. [16]), cells expressing wild-type S100P protein exhibited markedly stronger staining with intense foci of S100P in the cytoplasm and nuclear regions of the cells (Figure 1e). There were extensive clusters of NMMIIA foci, mainly at the cell periphery (Figure 1d), with only some colocalising with S100P, predominantly in the perinuclear region of the cells (Figure 1f). The foci of S100P staining in the wild-type-S100P-expressing Rama 37 cells observed here is similar to that observed previously in MCF-7 cells [19] using a glass substratum; in contrast, in the present experiments, the cells were grown on a fibronectin substratum. Thus, the punctate staining pattern has been observed in two different cell systems, grown on two different substrata. However, the strong nuclear staining observed in the MCF-7 cells was not seen in the present experiments. The structural basis of the punctate staining of wild-type S100P and how it might enhance cell migration in the MCF-7 cells [19] and in Rama 37 cells in the present experiments is unknown.

Although cells expressing the S100P mutant proteins (Figure 1g–l) exhibited less intense fluorescence than the wild-type protein, they did not exhibit to the same extent the localised foci of S100P or NMMIIA found in cells expressing wild-type protein. Instead, clear filamental structures of NMMIIA were observed at the cell periphery (Figure 1g,j) that resembled those observed in the control S100P-negative cells (Figure 1a). The proportion of K95A and ΔK95 mutant-S100P-protein-expressing cells that displayed a NMMIIA filamental structure resembling that of the S100P-negative control cells was not significantly different from the S100P-negative cells (K95A, *p* = 0.506; ΔK95, *p* = 0.818; Supplementary Table S2). It has been reported that S100P does not bind to NMMIIB [19]. Thus, it might be expected that the presence of S100P would not be associated with altered cytoskeletal arrangement of NMMIIB. The percentage of S100P-negative and S100P-expressing cells that exhibited the NMMIIB filamental structure found predominantly in the S100P-negative control cells was determined. The results showed that for cells expressing wild-type S100P or the K95A or ΔK95 mutant proteins, the percentage of cells expressing the myosin distribution characteristic of the S100P-negative cells was not significantly different from the S100P-negative control cells (*p* = 0.973, 0.919, and >0.9999, respectively; Supplementary Table S3). 

In S100P-negative, non-metastatic control cells, vinculin and paxillin were complexed into large foci at the ends of bundled actin filaments (Supplementary Figure S2). Cells expressing wild-type S100P exhibited a 4.3- or 3.5-fold reduction in the mean number of vinculin- or paxillin-positive focal adhesions per cell, respectively, compared to S100P-negative control cells (Table 2; both *p* < 0.0001). In contrast, cells expressing the K95A mutant S100P protein showed 2.1- and 1.8-fold higher numbers of vinculin and paxillin focal adhesions per cell, respectively, than wild-type S100P cells (vinculin and paxillin, both *p* < 0.0001) but 48% and 53% of those in the S100P-negative, control cells (Table 2; vinculin and paxillin, both *p* < 0.0001). ΔK95-mutant S100P protein exhibited mean numbers of vinculin and paxillin clusters per cell (Table 2) 5.1- and 4.5-fold higher, respectively, than cells expressing wild-type protein (both *p* < 0.0001) but, surprisingly, also 1.2- and 1.3-fold higher than the control cells (vinculin, *p* 0.0017; paxillin, *p* < 0.0001). These results suggest that mutation/deletion of the C-terminal lysine reduces the ability of S100P to alter the cytoskeletal organisation, a possible consequence of the reduced interaction of S100P with NMMIIA. 

### 3.3. The Effect of S100P Mutants on Transwell and Scratch-Wound Cell Migration

**Figure 2:**
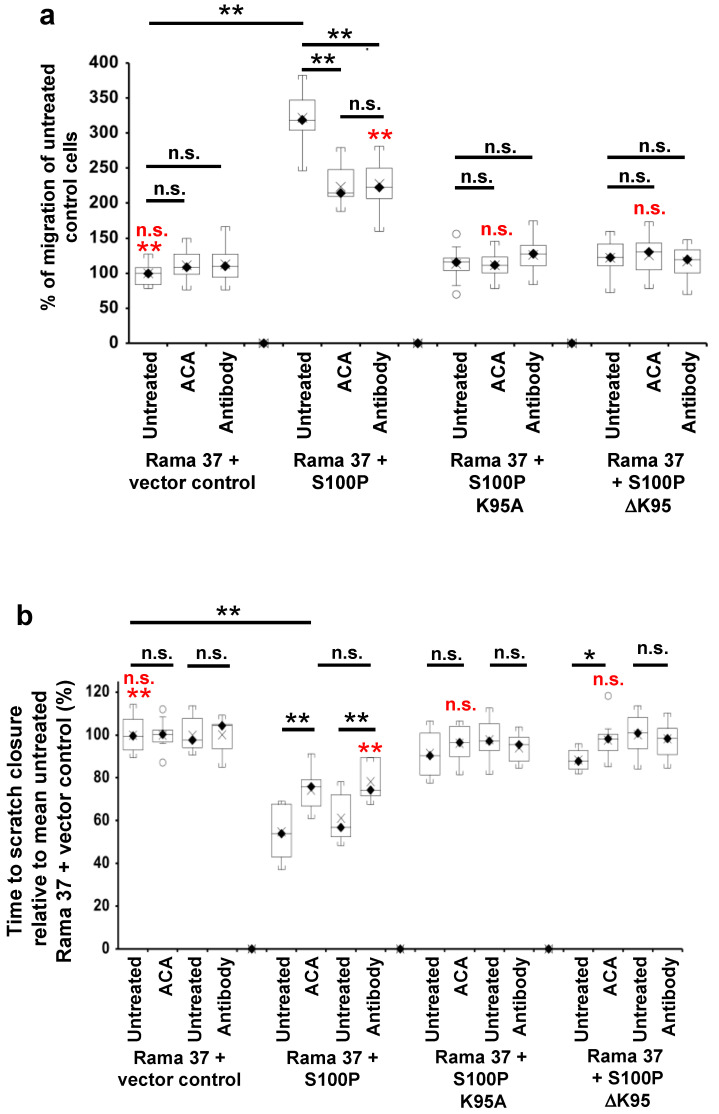

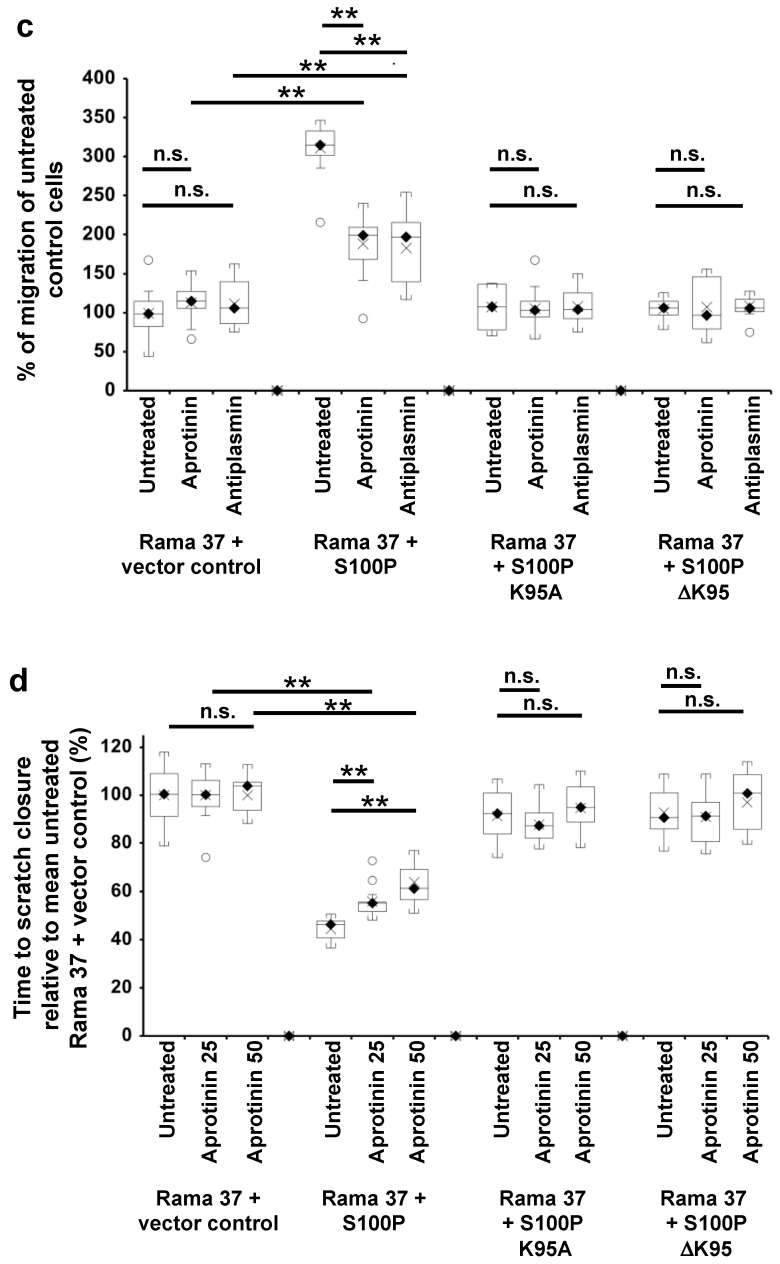

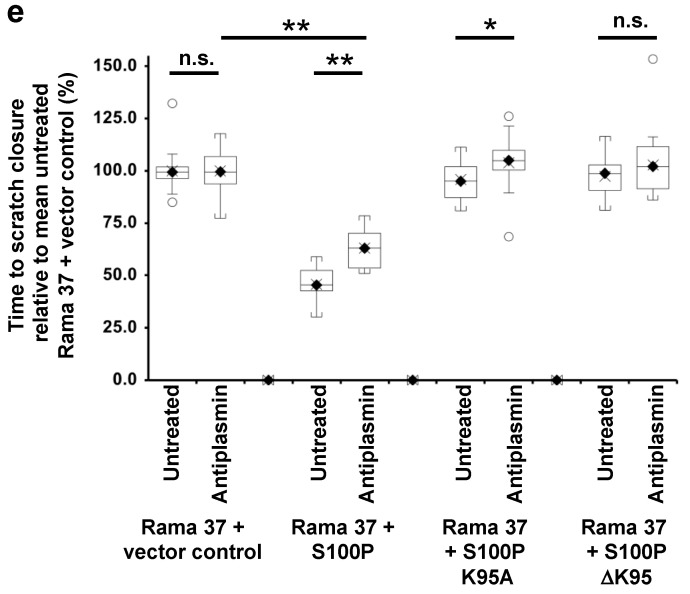
Effect of 6-aminocaproic acid, S100P antibody, aprotinin, or α-2-antiplasmin on migration of cells expressing wild-type and mutant S100P proteins. Transwell migration assays (**a**,**c**) or scratch migration assays in a Cell-IQ incubator (**b**,**d**,**e**) were carried out as described in Materials and Methods for cells expressing wild-type S100P (Rama 37 + S100P), K95A-mutant of S100P (Rama 37 + S100P K95A), ΔK95 mutant of S100P (Rama 37 + S100P ΔK95), or cells not expressing S100P (Rama 37 + vector control). In (**a**,**c**), the number of cells passing through to the underside of the membrane in 16 h were counted and are plotted as percentages of the mean number of untreated Rama 37 + vector control cells passing through the membrane. In (**b**,**d**,**e**), the times to scratch-wound closure are expressed as a percentage of the mean value for untreated Rama 37 + vector control cells. The effect on migration of cells of addition to the extracellular medium (upper and lower chambers in the case of Transwell assays) of 10 mM 6-aminocaproic acid (ACA, panels **a**,**b**) or of an R&D Systems polyclonal goat S100P-specific antibody (Cat No. AF2957) at a concentration of 0.2 ng/μL in the medium (Antibody, panels **a,b**) are shown. Each box and whisker plot shows the dispersion of data from 14 (**a**) or 11–12 (**b**) wells in two independent experiments carried out at different times. (**c**) Effect on Transwell migration of 50 μg/mL aprotinin (Aprotinin) or 10 μg/mL α-2-antiplasmin (Antiplasmin) in the extracellular medium. (**d**) Effect on scratch-wound migration of 25 μg/mL aprotinin (Aprotinin 25) or 50 μg/mL aprotinin (Aprotinin 50) in the extracellular medium. (**e**) Effect of 10 μg/mL α-2-antiplasmin (Antiplasmin) on scratch-wound migration. Each box and whisker plot shows the dispersion of data from 14 (**c**) or 14–25 wells (**d**,**e**) in two independent experiments carried out at different times. In all panels, *p*-values are indicated for comparison between the box and whisker plots beneath the ends of the horizontal lines as not significant (n.s., *p* > 0.05), or significant * (*p* between 0.001 and 0.05) or ** (*p* < 0.0001). For clarity, some *p*-value comparisons are shown without lines and in red colour, as follows: (**a**), n.s., Transwell assay Rama 37 + S100P K95A or Rama 37 +ΔK95 in the presence of 6-aminocaproic acid not significantly different from vector control (Dunnett post-hoc multiple comparison with a control); ** Rama 37 +S100P antibody significantly faster than Rama 37 + vector control, *p* < 0.0001; (**b**), scratch-wound assay, n.s. Rama 37 + S100P K95A or Rama 37 + ΔK95 in the presence of 6-aminocaproic acid not significantly different from vector control; ** Rama 37 +S100P antibody significantly faster than Rama 37 + vector control (*p* < 0.0001). In all boxes, the black diamond and line show the median value; the cross shows the mean value. The white circles outside the whiskers denote outliers of >1.5 times the interquartile range.

Given their reduction in metastatic potential and the key role of cell migration in metastasis [38], the effect of the C-terminal mutants on S100P-directed cellular migration was tested using two independent assays of cellular motility, Transwell migration [39], and scratch-wound closure [40,41] (Figure 2). In Figure 2, results of the Transwell migration assays are expressed as a percentage of the number of untreated, S100P-negative control cells passing through the membrane, whereas the results of the scratch migration assays are expressed as a percentage of the time-to-wound closure of untreated S100P-negative control cells (slower migration rate indicated by a longer time-to-wound-closure). Cells expressing wild-type S100P protein exhibited a 3-fold higher rate of Transwell migration than S100P-negative control cells transfected with empty vector (*p* < 0.0001, Figure 2a). In contrast, cells expressing the K95A or ΔK95 mutant S100P proteins exhibited Transwell migration rates that were reduced to 35 and 38%, respectively, of that of cells expressing wild-type S100P protein (K95A and ΔK95 both *p* < 0.0001) and not significantly 1.13- and 1.22-fold higher than S100P-negative, control cells (K95A, *p* = 0.695; ΔK95, *p* = 0.147; Figure 2a and Supplementary Figure S3).

Since migration rates in Transwell chambers are influenced by serum chemotaxis, directional cellular migration was tested using a scratch-wound assay. The wild-type S100P-expressing cells closed the scratch wound 1.7-fold faster than the S100P-negative control cells (*p* < 0.0001, Figure 2b and Supplementary Figure S4); however, cloned cells expressing the K95A or ΔK95 mutant S100P proteins closed the scratch wound 63% and 62.5%, respectively, slower than cells expressing wild-type S100P (K95A and ΔK95, both *p*< 0.0001; Figure 2b and Supplementary Figure S4) and just 1.06- and 1.06-fold faster than S100P-negative, control cells (K95A, *p* = 0.172; ΔK95, *p* = 0.126; Figure 2b). These results show that the presence of the C-terminal lysine of S100P is associated with the S100P-enhanced cell migration.

### 3.4. Effect of 6-Aminocaproic Acid or S100P Antibody on the Migration of Rama 37 Cells Expressing Wild-Type or Mutant S100P Proteins 

S100P enhances plasminogen activation by tissue plasminogen activator in a C-terminal lysine dependent manner and the activation is competed by the lysine analogue, 6-aminocaproic acid (6-ACA) [16]. Thus, in order to examine how the C-terminal lysine of S100P affects cell migration, 6-ACA was added to the medium of the cloned cell lines expressing wild-type or mutant S100P proteins or control S100P-negative cells, and its effect on cell migration was tested using Transwell and scratch-wound assays (Figure 2). The results of the Transwell migration assays are expressed as a percentage of the number of untreated, S100P-negative control cells passing through the membrane, whereas the results of the scratch migration assays are expressed as a percentage of the time-to-wound-closure of untreated S100P-negative control cells (slower migration rate indicated by a longer time-to-wound-closure).

The addition of 10 mM 6-ACA to the medium of S100P-negative control cells had no effect on their migration in either Transwell migration (Figure 2a and Supplementary Figure S3; *p* = 0.248) or scratch-wound assay (Figure 2b and Supplementary Figure S4; *p* = 0.999). However, in cells expressing wild-type S100P, 6-ACA significantly reduced motility in the Transwell migration assays by about 30% (Figure 2a and Supplementary Figure S3; *p* < 0.0001) and increased scratch-wound closure time by 35.6% (Figure 2b and Supplementary Figure S4; *p* < 0.0001), but, importantly, for the Transwell assay, the migration rate was still 2.2-fold higher (Figure 2a and Supplementary Figure S3; *p* < 0.0001), and for the scratch assay, time-to-wound-closure was still 25.7% shorter (Figure 2b and Supplementary Figure S4; *p* < 0.0001) than for S100P-negative control cells. Thus, the lysine analogue, 6-ACA at concentrations that effectively compete lysine-dependent activities [42] abolishes only ~50% of the wild-type S100P-dependent increase in two separate assays of cell migration. 

6-ACA had no effect on Transwell migration of cells expressing either K95A or ΔK95 C-terminal mutant proteins (Figure 2a and Supplementary Figure S3; K95A, *p* = 0.843; ΔK95, *p* = 0.72). In the scratch-wound assay, 6-ACA had no effect on cells expressing the K95A mutant protein (*p* = 0.207) and a small (10.5%) inhibitory effect (*p* = 0.013) on the migration of cells expressing the ΔK95 mutant protein (Figure 2b and Supplementary Figure S4). However, as with the Transwell assay, the resulting rates of wound closure with 6-ACA were not different from the S100P-negative control cells (Figure 2b and Supplementary Figure S4; K95A, *p* = 0.963; ΔK95, *p* = 0.9996). Thus, in both assays of migration, the absence of the C-terminal lysine reduces the effect of 6-ACA on cell migration.

In addition to its lysine competitive activity, which might not be entirely specific for the C-terminal lysine, 6-ACA has been reported to be taken up into cells, at least in a clinical situation [43,44], and to affect plasmin activity [45]. Thus, to rule out these possibilities and to establish the extracellular location of the inhibitory effect of 6-ACA and to investigate the involvement of activated plasmin, more-specific inhibitors and their effects on cell migration were tested.

**Figure 3:**
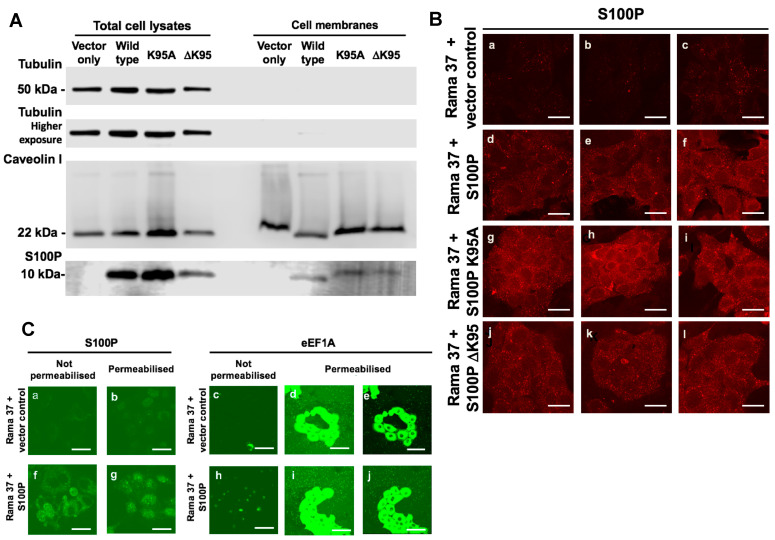
Association of S100P with cell membranes. (**A**) Total cell extracts and membrane fractions prepared as described in Materials and Methods from Rama 37 cells expressing S100P proteins and were subjected to Western blotting to detect S100P protein, the cytoplasmic marker, tubulin, and the plasma membrane marker, caveolin 1. (**B**) Living cells not expressing S100P (Rama 37 + vector control; panels **a**–**c**) or expressing wild-type S100P (Rama 37 + S100P; panels **d**–**f**), K95A mutant S100P (Rama 37 + S100P K95A; panels **g**–**i**), or S100P protein with the C-terminal amino acid deleted (Rama 37 + S100P ΔK95; panels **j**–**l**) were incubated with S100P antibody in the culture medium, and bound antibodies were detected with Cy-3-labelled secondary antibody, as described in Materials and Methods and observed with a Zeiss LSM510 confocal laser scanning microscope. Three separate typical fields are shown for each cell line. (**C**) Antibodies directed at S100P (panels **a**,**f**) or cytoplasmic protein, eEF1A (panels **c**,**h**), were added to the culture medium of living S100P-negative Rama 37 cells (Rama 37 + vector control; panels **a**,**c**) or S100P-positive Rama 37 cells (Rama 37 + S100P; panels **f**,**h**). Parallel cultures of S100P-negative cells (Rama 37 + vector control; panels **b**,**d**,**e**) and S100P-positive cells (Rama 37 + S100P; panels **g**,**i**,**j**) were permeabilised before being treated with the antibodies directed at S100P (permeabilised; panels **b**,**g**) or eEF1A (permeabilised; panels **d**,**e**,**i**,**j**). Following fixation, bound antibodies were detected with fluorescein isothiocyanate-conjugated secondary antibodies (Materials and Methods) and observed with a Zeiss LSM510 confocal laser scanning microscope. Panels (**e**,**j**) show the same fields as panels (**d**,**i**), but the intensity of the fluorescence signal has been reduced to show individual cells. Bars, (**B**,**C**) = 50 μm.

When an S100P antibody was present at a concentration of 0.2 ng/μL in the medium during migration assays, the migration of S100P-negative cells was not affected in either the Transwell (Figure 2a and Supplementary Figure S3; *p* = 0.187) or scratch-wound assays (Figure 2b and Supplementary Figure S4; *p* = 0.983); however, the migration of wild-type S100P-expressing cells was reduced by ~30% in the Transwell assays (Figure 2a and Supplementary Figure S3; *p* < 0.0001), and there was a 28% increase in time-to-wound-closure (Figure 2b and Supplementary Figure S4; *p* < 0.0001) compared to untreated S100P-expressing cells. As with 6-ACA, importantly, S100P-dependent migration was only ~50% reduced, with the antibody-treated cells still migrating 2-fold faster than control, S100P-negative cells in the Transwell (Figure 2a and Supplementary Figure S3; *p* < 0.0001) and 22% faster in the scratch-wound assays (Figure 2b and Supplementary Figure S4; *p* < 0.0001). Furthermore, the reduction in migration with extracellular antibody was not different from that observed with 6-ACA in both the Transwell (Figure 2a and Supplementary Figure S3; *p* = 0.7) and scratch-wound assays (Figure 2b and Supplementary Figure S4; *p* = 0.299). The presence of S100P antibody in the medium did not change the migration of cells expressing the K95A or ΔK95 mutant proteins in the Transwell (Figure 2a and Supplementary Figure S3; K95A, *p* = 0.185; ΔK95, *p* = 0.537) or scratch-wound assays (Figure 2b and Supplementary Figure S4; K95A, *p* = 0.306; ΔK95, *p* = 0.586), even though the antibody detected both wild-type and mutant S100P proteins in Western blots (Figure 3A). These results suggest that S100P exposure from outside the cell contributes ~50% of the S100P-dependent increased migration in both Transwell and wound-closure assays but not when S100P lacks the C-terminal lysine residue.

### 3.5. Effect of Plasmin Inhibitors on the Migration of Rama 37 Cells Expressing Wild-Type and Mutant S100P Proteins 

The effects on cell migration of two plasmin inhibitors, aprotinin and α-2-antiplasmin, were examined. Fifty μg/mL aprotinin or 10 μg/mL α-2-antiplasmin reduced the migration rates of wild-type S100P-expressing cells in the Transwell assay by 40% and 41%, respectively, compared to untreated cells (Figure 2c and Supplementary Figure S3; aprotinin; α-2-antiplasmin, both *p* < 0.0001). In the scratch-wound assay, time-to-wound-closure increased 25.4% for 25 μg/mL aprotinin (*p* = 0.001), 43.5% for 50 μg/mL aprotinin (*p* < 0.0001), and 37.8% for 10 μg/mL α-2-antiplasmin (*p* < 0.0001) (Figure 2d,e and Supplementary Figure S4). However, as with 6-ACA and S100P antibody, the cells treated with plasmin inhibitors still exhibited migration rates that were greater than S100P-negative control cells in Transwell (Figure 2c and Supplementary Figure S3; 50 μg/mL aprotinin, 1.9-fold greater; α-2-antiplasmin, 1.8-fold greater, both *p* < 0.0001) and scratch-wound assays (Figure 2d and Supplementary Figure S4; aprotinin, 25 μg/mL, 44% greater, 50 μg/mL, 36% greater; Figure 2e and Supplementary Figure S4, α-2-antiplasmin, 37% greater, all *p* < 0.0001). Neither aprotinin nor α-2-antiplasmin had any effect on the migration of the S100P-negative, control cells in either assay (Transwell assay, Figure 2c and Supplementary Figure S3; 50 μg/mL aprotinin, *p* = 0.24; α-2-antiplasmin, *p* = 0.3; scratch-wound assay, Figure 2d and Supplementary Figure S4; 25 μg/mL aprotinin, 50 μg/mL aprotinin and α-2-antiplasmin, all *p* > 0.9999) nor on cells expressing the mutant proteins in either assay (Transwell assay, Figure 2c and Supplementary Figure S3; K95A, 50 μg/mL aprotinin, *p* = 0.824; α-2-antiplasmin, *p* = 0.947; ΔK95, 50 μg/mL aprotinin, *p* = 0.848; α-2-antiplasmin, *p* = 0.742; scratch-wound assay, Figure 2d and Supplementary Figure S4; K95A, 25 μg/mL or 50 μg/mL aprotinin, *p* = 0.31; ΔK95, 25 μg/mL or 50 μg/mL aprotinin, *p* = 0.237; α-2-antiplasmin, Figure 2e and Supplementary Figure S4; ΔK95, *p* = 0.072). However, α-2-antiplasmin inhibited cells expressing the K95A mutant protein by a small but significant 8.3% (Figure 2e and Supplementary Figure S4, *p* = 0.01). Since only plasmin is inhibited by both aprotinin [46] and α-2-antiplasmin [47], and since similar reductions in the migration rates of the wild-type S100P-expressing cells by these two inhibitors are observed in both the Transwell assays (Figure 2c and Supplementary Figure S3; 50 μg/mL aprotinin, 40%; α-2-antiplasmin, 41%) and scratch-wound assays (Figure 2d,e and Supplementary Figure S4; 50 μg/mL aprotinin, 43.5%; α-2-antiplasmin, 37.8%), the results strongly suggest that plasmin activity contributes but only in part to the S100P-enhanced migration in the wild-type S100P-expressing Rama 37 cells. 

The number of focal adhesions in S100P-expressing cells was not increased by the addition of 6-aminocaproic acid (vinculin *p* = 0.614, paxillin *p* = 0.488), the S100P antibody (vinculin *p* = 0.269, paxillin *p* = 0.972), aprotinin (paxillin, *p* = 0.19), or α-2-antiplasmin (paxillin, *p* = 0.4) (Supplementary Tables S4 and S5), strongly suggesting that the S100P/plasmin pathway does not involve alteration in the numbers of focal adhesions per cell, clearly distinguishing this pathway from that initiated by binding of S100P to NMMIIA. 

### 3.6. Membrane-Associated S100P 

A membrane fraction containing the cell surface marker Caveolin 1 was isolated by sucrose density gradient centrifugation (Materials and Methods). This membrane fraction contained almost undetectable levels of the cytoplasmic marker, tubulin (Figure 3A) but clearly contained S100P in cells expressing wild-type, K95A, or ΔK95 mutant S100P proteins. S100P was undetectable in S100P-negative, vector-transfected cells (Figure 3A). Quantitation of Western blots of caveolin-containing membrane preparations and total cell lysates from two independent experiments showed that for cells expressing wild-type S100P, K95A, and ΔK95 mutant S100P proteins, a mean of 2.2% ± SD 0.95%, 1.7% ± 0.94%, and 3.6% ± 1.0% of the total S100P in cell lysates, respectively, was detected in the membrane fractions. The membrane levels of the K95A and ΔK95 mutant proteins were both 71% of the membrane level of the wild-type S100P protein. Direct analysis of the presence of S100P at the external cell membrane was also assessed using immunofluorescence staining of unpermeabilised living cells by S100P antibody added to the culture medium. An S100P signal was observed on the surface of cells expressing wild-type S100P (Figure 3B(d–f)), K95A (Figure 3B(g–i)), and ΔK95 (Figure 3B(j–l)) mutants but not on the S100P-negative control cells (Figure 3B(a–c)). Staining with antibody to the intracellular protein, eEF1A was virtually absent in unpermeabilised living cells (Figure 3C(c,h)) in contrast to the strong staining observed with permeabilised cells (Figure 3C(d,e,i,j)), showing that the surface staining for S100P (Figure 3B(d–f) and Figure 3C(f)) was not due to cell lysis. These results suggest that a similar small proportion of the cellular wild-type or mutant S100P was isolated with the cells’ membranes and show that the failure of the mutant proteins to support migration is not mainly due to a lack of their association with the cells’ membranes. 

### 3.7. The Effect of Extracellularly Added S100P on the Migration of S100P-Negative Control Cells

**Figure 4:**
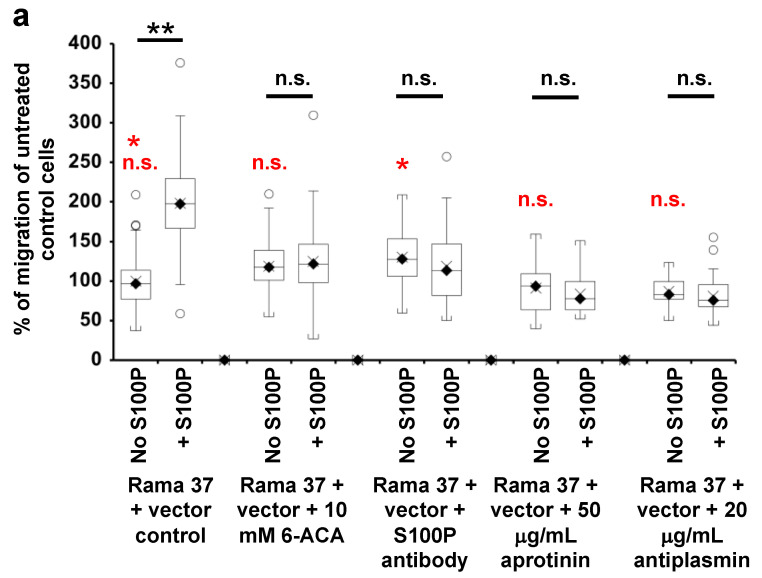

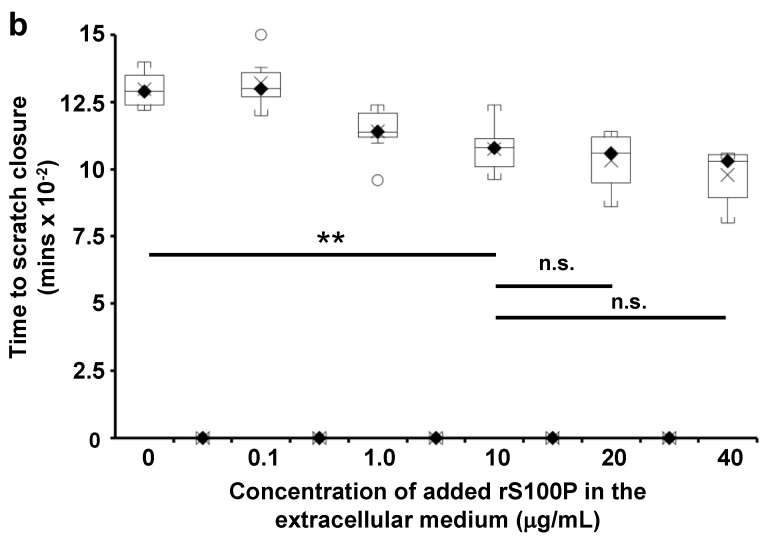
Effect of extracellular addition of S100P on migration of S100P non-expressing cells in Transwell and scratch-wound migration assays. (**a**) Transwell migration assays were carried out as described in Materials and Methods. The number of S100P-negative Rama 37 empty-vector-transfected control cells passing through to the underside of the membrane in 16 h in the presence of 10 mM 6-ACA (Rama 37 + vector + 10 mM 6-ACA), R&D Systems polyclonal goat S100P-specific antibody, Cat. No AF2957 at a concentration of 0.2 ng/μL in the medium (Rama 37 + vector + S100P antibody), 50 μg/mL aprotinin (Rama 37 + vector + 50 μg/mL aprotinin), or 20 μg/mL α-2-antiplasmin (Rama 37 + vector + 20 μg/mL antiplasmin), either in the absence (No S100P) or in the presence (+ S100P) of 10 μg/mL recombinant S100P (rS100P) were counted and are plotted as a percentage of the mean of untreated Rama 37 + vector control cells (Rama 37 + vector control). Each box and whisker plot shows the dispersion of data from 60 separate data points from 4 independent experiments (control, 6-ACA, S100P antibody) or 10 to 30 data points from 2 independent experiments (aprotinin and antiplasmin) carried out at different times. (**b**) Rama 37 cells transfected with empty expression vector were subjected to scratch-wound assays in the absence of or in the presence of various concentrations of rS100P added to the extracellular medium and the time-to-scratch-closure recorded for 9 separate wells in two independent experiments carried out at different times. For both (**a**,**b**), *p*-values are indicated for comparison between the box and whisker plots beneath the ends of the horizontal lines as n.s. not significant (*p* > 0.05) or significant ** (*p* < 0.0001). In (**a**), for clarity, some *p*-value comparisons are shown without lines and in red colour, as follows: n.s., no-S100P controls for 10 mM 6-ACA, 50 μg/mL aprotinin, and 20 μg/mL α-2-antiplasmin are not significantly different from the no-S100P, no-addition control (*p* = 0.055, 0.946, 0.932, respectively, Dunnett post-hoc test), or * no S100P for 0.2 ng/μL S100P antibody, significantly faster than the no-S100P, no-addition control (*p* = 0.0002). In all boxes for (**a**,**b**), the black diamond and line show the median value; the cross shows the mean value. The white circles outside the whiskers denote outliers of >1.5 times the interquartile range.

To confirm that S100P can alter cell migration from outside the cells, recombinant (r)S100P was added at 10 μg/mL to the medium of S100P-negative empty vector control cells during migration assays, a concentration that activates plasminogen activators in vitro [16]. Addition of rS100P to the medium resulted in a 2-fold increase in migration in the Transwell assay (Figure 4a; *p* < 0.0001), less than the ~3-fold increase resulting from endogenously expressed S100P protein and an 18.6% decrease in time-to-wound-closure (Figure 4b; *p* < 0.0001). In the scratch-wound assay, increasing the extracellular concentration of rS100P to 20 or 40 μg/mL did not reduce the time-to-wound-closure observed with 10 μg/mL (*p* = 0.583 and 0.11, respectively, Figure 4b), strongly suggesting that increasing the concentration of rS100P any further does not increase cell migration. Amounts of 10 mM 6-ACA, S100P antibody, 50 μg/mL aprotinin, or 20 μg/mL α-2-antiplasmin abolished the extracellular recombinantly-added, S100P-dependent increase in cell migration to a level not different from migration in the absence of added S100P (Figure 4a; 6-ACA, *p* = 0.41; anti-S100P antibody, *p* = 0.098; aprotinin, *p* = 0.483; α-2-antiplasmin, *p* = 0.695). In contrast, in the absence of added rS100P, none of these inhibitors significantly reduced cell migration (Figure 4a; 6-ACA, *p* = 0.055; anti-S100P antibody, significant increase, *p* = 0.0002; aprotinin, *p* = 0.946; α-2-antiplasmin, *p* = 0.932). The results show that 6-ACA, aprotinin, S100P antibody, and α-2-antiplasmin each abolished completely the cell migration resulting from S100P outside the cell, with no inhibitory effect on S100P-independent cell migration. 

## 4. Discussion

For the first time, it is shown here that removing or replacing the single C-terminal lysine residue of S100P protein with alanine significantly reduces its ability to promote metastasis in a well-established animal model [23]. In this animal model metastasis is predominantly to lungs and, over a longer time frame, to liver. The present experiments were terminated after 2 months, so liver metastases were not observed. Human breast cancer also metastasises eventually to the bony skeleton. Although most of the changes in the C-terminal lysine showed a significant reduction in metastasis, the pool of K95A S100P transfectants did not, suggesting that more rats than the 19 used were needed to achieve statistical significance. However, the decreases in metastasis were closely mimicked by decreases in Transwell/scratch-wound migration and increases in focal adhesions/cell, suggesting that these processes are all linked and that these in-vitro processes may be used to substitute for metastasis assays in vivo. 

The K95A and ΔK95 mutants of S100P have been shown previously to abolish S100P invasion through Matrigel in a Transwell invasion assay [16]. Thus, the novel finding is now made that cells expressing C-terminal mutant S100P proteins exhibit reduced cell migration, the major driver of metastasis in this system [38]; increased actin/myosin organisation; and increased numbers of vinculin/paxillin focal adhesions when compared to cells expressing wild-type S100P. It is possible that the changes in focal adhesion numbers represent changes in focal adhesion turn-over rate; however, separate experiments in which S100P-expressing cells were gently removed from the substratum using hydrostatic pressure without detergent showed approximately the same numbers of focal adhesions in S100P-positive and S100P-negative cells. When the residue on the substratum was subsequently treated with non-ionic detergents, the loss in the S100P-positive cells was then observed [48]. This result suggests that it is the more stable, non-ionic-detergent-resistant focal adhesions that are lost in the presence of S100P. 

In other model systems, S100P has been reported to affect metastasis or processes associated with metastasis. In pancreatic cell lines, the S100-protein-binding drug, cromolyn, reduced the size of metastases derived from BxPC3, Mpanc 96, and Panc-1 cells injected into immunocompromised mice [49]. Similarly, it has been shown that receptor for advanced glycation end products (RAGE) antagonist peptide (RAP) inhibited interaction of S100P with this extracellular receptor and reduced not only growth and migration but also reduced activation of NFκB. Furthermore, RAP reduced metastasis in vivo of pancreatic tumours in immunocompromised mice, suggesting a role for RAGE in S100P-associated metastasis [50]. However, in these immunocompromised systems, S100P affected cell/tumour growth in contrast to the syngeneic, immunocompetent, mammary system of the present experiments in which the S100P mutants did not affect tumour incidence. In breast cancer cell lines, it has been reported that the long non-coding RNA, NORAD, sequesters S100P, and its reduction in breast cancer cells allows S100P to exert its prometastatic roles [51]. However, such an upstream activation process does not affect the results presented here on the downstream mechanisms of metastatic activity of S100P. 

S100 proteins act intracellularly by interacting with partner proteins [52]; however, the interaction of S100P with its major targets, ezrin [17] and IQGAP [18], are not affected by deletion of some of the C-terminal amino acid residues of S100P [18,20]. S100P binds to the RAGE receptor on the cell surface [15]. The hydrophobic binding patch on calcium-bound S100P responsible for this interaction includes G93 in the potentially unstructured C-terminal region of human S100P [15]. S100P has been shown to co-localise with NMMIIA and to interact with the S100-binding region of NMMIIA in living cells using fluorescence lifetime imaging [19]. The failure of the C-terminal lysine mutants to enhance cell migration in the present experiments is likely to be associated with the observed 10-fold reduction in interaction between S100P C-terminal mutant proteins and NMMIIA in vitro. Since S100P is phylogenetically closely related to S100B, it is also possible that the interaction of S100P with NMMIIA follows a two-step interaction model in which the C-terminus strengthens the target interaction, as has been proposed for the interaction of S100B with its targets [53]. The precise involvement of the C-terminal lysine of S100P in its interaction with NMMIIA will only become evident on determination of the full, three-dimensional structure of the complex of S100P with NMMIIA. However, the determination of the three-dimensional structure of S100A4 in its complex with a peptide consisting of the binding site of human NMMIIA did not identify a direct role for the two C-terminal lysines of S100A4 in its stable complex with the NMMIIA sequence [13]. Furthermore, for S100A4, it has been suggested separately that the charged C-terminal lysines prevent binding of its C-terminal region to the target-binding, hydrophobic regions exposed upon calcium activation within an S100A4 dimer [54]. For S100P, the ΔK95 mutant possesses a C-terminal hydrophobic leucine residue and the replacement K95A mutant, an alanine residue with a smaller hydrophobic sidechain, in both cases these alterations may possibly lead to hydrophobic interaction between the modified C-terminus and the hydrophobic core in an S100P dimer in the calcium-activated state. Such intramolecular blocking of the myosin binding sites may account for the reduction in myosin binding, consequent changes in the numbers of focal adhesions, reduction in myosin-associated cell migration, and metastasis. The S100P ΔK95 mutant was more effective at reducing the metastatic potential of the cells (Table 1), at restoring an S100P-negative filamental pattern of NMMIIA (Supplementary Table S2), and at restoring the presence of focal adhesions than the K95A mutant protein (Table 2), observations which reinforce the link between cytoskeletal changes and metastatic potential in these cells. Thus, differences in the hydrophobicity of the C-terminal leucine of the ΔK95 mutant and alanine of the K95A mutant may account for the observed weaker binding to myosin, higher number of focal adhesions, and larger reduction in metastasis observed with the ΔK95 mutant than with the K95A mutant. This mechanism provides a possible explanation for the dramatic consequences of C-terminal deletion on S100P function, since structural studies have failed to show a direct mechanistic role for the flexible C-terminal region of S100P [11] or S100A4 [13,55]. However, it should be noted that the three-amino-acid residues of the C-terminal region beyond helix 4 in calcium-bound S100P is much shorter than the eight residues of S100A4 [11], and thus, the C-terminal region of S100P may not behave in the same way as that of S100A4 upon C-terminal lysine removal.

The signalling pathways by which NMMIIA-interacting S100 proteins, such as S100P [19] or S100A4 [13], alter the numbers of focal adhesions is not presently known. Nor is it known how the reduced binding to NMMIIA of S100P arising from the C-terminal mutants (Supplementary Table S1 and Figure S1) might affect other actomyosin signalling pathways. 

A second novelty of the present findings is the identification, using inhibitors, of a second pathway by which S100P promotes cell migration in a cellular system of S100P-driven metastasis. This second migration pathway is associated with plasmin protease activity and does not involve changes in the focal adhesion complexes of cells (Supplementary Tables S4 and S5). The presence of this second, NMMIIA-independent pathway, is suggested by previous experiments in HeLa cells, showing that upon knockdown of NMMIIA with specific siRNAs, there was still residual stimulation of cell migration due to S100P (Figure 3A of [19]). How plasmin may induce cell migration associated with metastasis remains to be determined. In keratinocytes, extracellular plasmin increases chemotaxic but not chemokinetic migration [56]; in human bronchial epithelial cells, plasmin activates MMP-9 to enhance wound closure [57], and in endothelial cells, plasmin binds to cell-surface integrin, αvβ3 [58], or to integrin α9β1 in CHO cells [59]. Plasmin can release specific molecules from the extracellular matrix, such as cysteine-rich 61 protein, which supports endothelial cell migration [60], or CCL21, which supports migration of dendritic and T cells of the immune system [61].

Since in the present experiments, the plasmin pathway can be inhibited by an S100P-specific antibody added to the cellular medium and because antibodies do not cross cell membranes [62], this pathway is likely associated with S100P exposure to the extracellular medium, unlike the myosin pathway above, which is driven by intracellular S100P. It is well established that the C-terminal lysine of the related protein, S100A10, activates plasminogen activation processes by being displayed on the outside of the cell surface [63]. The dimeric S100A10 protein constitutes two subunits of the annexin A2 tetramer [63], which associates with the cell membrane and displays the C-terminal lysines of S100A10 to the external milieu, where they activate tissue plasminogen activator to convert plasminogen to plasmin. A similar association between annexin A2 and S100A4 has also been proposed [64]. However, it has not been possible yet to demonstrate interaction between S100P and annexin A2 [16]. Alternatively, it has been reported that secreted S100P protein interacts with the RAGE receptor at the cell surface [15], where plasmin activation events could take place. However, how S100P might leave the cell without the presence of a secretory signal is not presently known.

The completely novel finding in the present work is the demonstration, in a well-established model system of metastasis [3,23,24,25], of two concurrent pathways that activate cell migration, a major driver in metastatic cells [38]. On the one hand, the presence of the C-terminal lysine maintains the ability of S100P to interact with and inhibit the predominant cell-migration-inhibitory activity of NMMIIA through an intracellular focal adhesion pathway; this interaction then enhances cell migration [19]. On the other hand, the C-terminal lysine also enables extracellular activation of plasminogen activation processes, which are now shown to contribute to cell migration through a focal-adhesion-independent mechanism. However, the precise molecular intermediates that link plasminogen activation and cell migration in this system remain to be determined. While these results suggest two separate pathways, one associated with changes in the numbers of focal adhesions and one that is not, it is not possible to rule out overlap of the two pathways at the level of any intracellular signalling induced by the activation of plasminogen. The results here show how S100P can affect more than one pathway of migration in a previously well-characterised cell system of metastasis [3,23] and identifies the C-terminus of S100P as a potential target for simultaneously inactivating such multiple pathways of S100P-driven metastasis.

## Figures and Tables

**Table 1:** Incidences of tumours and metastases.

Transfected DNA (Designation of Cell Line) ^a^	Incidence of Mammary Tumours (%) ^b^	*p*-Value ^c^	Incidence of Metastasis (%) ^d^	*p*-Value ^e^
None. Untransfected ^f^	18/20 (90)		0/18	
Rama 37 Vector only ^f^				
Clone	19/20 (95)		0/19 (0)	
Pool	23/23 (100)		2/23 (9)	
S100P wild-type				
Clone	27/27 (100)		19/27 (70)	
Pool	20/20 (100)		15/20 (75)	
K95A S100P				
Clone	21/21 (100)	*p* > 0.9999	7/21 (33)	*p* = 0.019
Pool	19/19 (100)	*p* > 0.9999	9/19 (47)	*p* = 0.105
ΔK95 S100P				
Clone	19/19 (100)	*p* > 0.9999	3/19 (16)	*p* = 0.0003
Pool	19/21 (90)	*p* > 0.9999	2/19 (11)	*p* < 0.0001

^a^ Nomenclature—S100P wild type, cells expressing non-mutant S100P protein; K95A S100P, cells expressing S100P with C-terminal lysine changed to alanine; ΔK95 S100P, cells expressing S100P with C-terminal lysine deleted; clone, a cell line derived from a single colony of transfected cells; pool, an uncloned pool of transfected cells; experimental unit, clone or pool of transfected cells. ^b^ Number of tumours/number of animals inoculated. Animals were randomised before use to ensure individual observations were independent for statistical analysis, encrypted, and hence blinded to the recorder of macroscopic and histological results to avoid bias. No adverse effects reported. ^c^ *p*-values by standard 2-sided Fisher’s Exact test for tumour incidences in vivo of a clone or a pool of transfected cells expressing K95A or ΔK95 mutant S100P proteins compared with a clone or a pool of transfectant cells expressing wild-type S100P protein. ^d^ Numbers of animals (%) with lung metastases/numbers of animals with tumours. ^e^ *p*-values by standard 2-sided Fisher’s Exact test for incidences of metastasis in vivo of a clone or a pool of transfected cells expressing K95A or ΔK95 mutant S100P proteins compared with a clone or a pool of transfectant cells expressing wild-type S100P protein. ^f^ For purpose of comparison only, the results for Rama 37 cells and vector are taken from Ismail, T. et al. [27], with permission of Oxford University Press.

**Table 2:** Quantitation of focal adhesions in cell lines with and without metastatic potential.

Cell Clone	Focal Vinculin ^a^			Focal Paxillin ^a^		
	No of Cells Counted	Mean Focal Adhesions/ Cell ± SD ^b^	Mean Focal Adhesions as % of Vector Control	No of Cells Counted	Mean Focal Adhesions/ Cell ± SD ^b^	Mean Focal Adhesions as % of Vector Control
Vector control	52	16.8 ± 4.0	100	54	15.2 ± 5.4	100
Wild-type S100P	51	3.9 ± 2.9 *	23.2	53	4.4 ± 3.1 **	28.9
K95A-mutant S100P	52	8.1 ± 4.7 ^¶^	48.2	51	8.0 ± 4.2 ^¶¶^	52.6
ΔK95-mutant S100P	51	19.8 ± 6.2 ^§^	117.9	50	20.0 ± 5.2 ^§§^	131.6

^a^ Cloned cell lines were stained for either vinculin or paxillin as described in Materials and Methods. Vinculin or Paxillin-stained focal adhesions were counted in about 50 cells from three independent experiments and the mean and standard deviation (SD) of the number per cell were calculated. b Significance of difference between 2 variables identified was calculated using Mann–Whitney U-test. * Significantly fewer vinculin focal adhesions than S100P-negative vector clone control cells (*p* < 0.0001). ** Significantly fewer paxillin focal adhesions than S100P-negative vector clone control cells (*p* < 0.0001). ^¶^ Significantly more vinculin focal adhesions than cell-clone-expressing wild-type S100P (*p* < 0.0001) but significantly fewer than S100P-negative vector clone control cells (*p* < 0.0001) and cell-clone-expressing ΔK95 mutant S100P (*p* < 0.0001). ^¶¶^ Significantly more paxillin focal adhesions than cell-clone-expressing wild-type S100P (*p* < 0.0001) but significantly fewer than S100P-negative vector clone control cells (*p* < 0.0001) and cell-clone-expressing ΔK95 mutant S100P (*p* < 0.0001). ^§^ Significantly more vinculin focal adhesions than cell-clone-expressing wild-type S100P (*p* < 0.0001) and significantly more than S100P-negative vector clone control cells (*p* = 0.0017). ^§§^ Significantly more paxillin focal adhesions than cell-clone-expressing wild-type S100P (*p* < 0.0001) and significantly more than S100P-negative vector clone control cells (*p* < 0.0001).

## Data Availability

Data supporting this publication are available from the corresponding authors.

## Supplementary Materials

The following are available online at https://www.mdpi.com/article/10.3390/biom11101471/s1, Figure S1: Kinetics of binding of mutant S100P proteins to immobilised recombinant C-terminal region of non-muscle myosin heavy chain, isoform A (rNMMIIA); Figure S2: Immunofluorescence localisation of vinculin/paxillin focal adhesions and actin filaments in transfected cells; Figure S3: Representative images for Transwell migration data for Figure 2; Figure S4: Representative images of scratch migration data for Figure 2; Table S1: Kinetics of binding of wild-type and mutant recombinant S100P proteins to immobilised recombinant C-terminal fragment of non-muscle myosin II heavy chain; Table S2: Quantitation in S100P-expressing cells of the myosin A fibre pattern that predominates in S100P-negative Rama 37 cells; Table S3: Quantitation in S100P-expressing cells of the myosin B fibre pattern that predominates in S100P-negative Rama 37 cells; Table S4: Effect of 6-aminocaproic acid and S100P antibody on the number of vinculin- and paxillin-stained focal adhesions in S100P-negative and wild-type S100P-expressing cells; Table S5: Effect of aprotinin and α-2-antiplasmin on the number of paxillin-staining focal adhesions in S100P-negative and wild-type S100P-expressing cells.
