# Supplementary material for: The Role of the C-Terminal Lysine of S100P in S100P-Induced Cell Migration and Metastasis
Source: Biomolecules. 2021 Oct 6;11(10):1471. doi: 10.3390/biom11101471 (PMC8533620; doi:10.3390/biom11101471)
Supplement: Supplementary file 1 [file biomolecules-11-01471-s001.zip › biomolecules-1398088-supplementary.pdf]

Wild type S100P

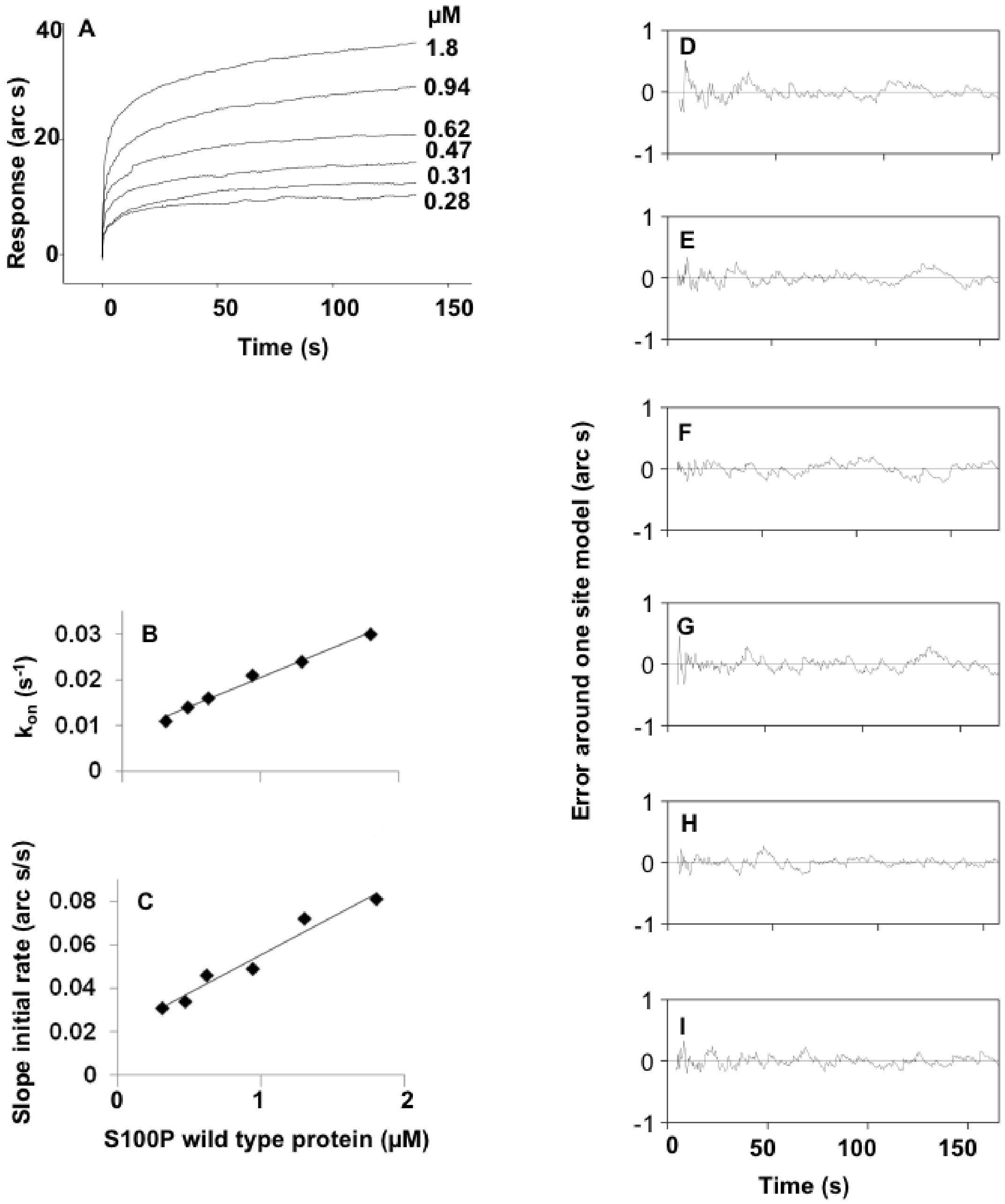

K95A mutant S100P

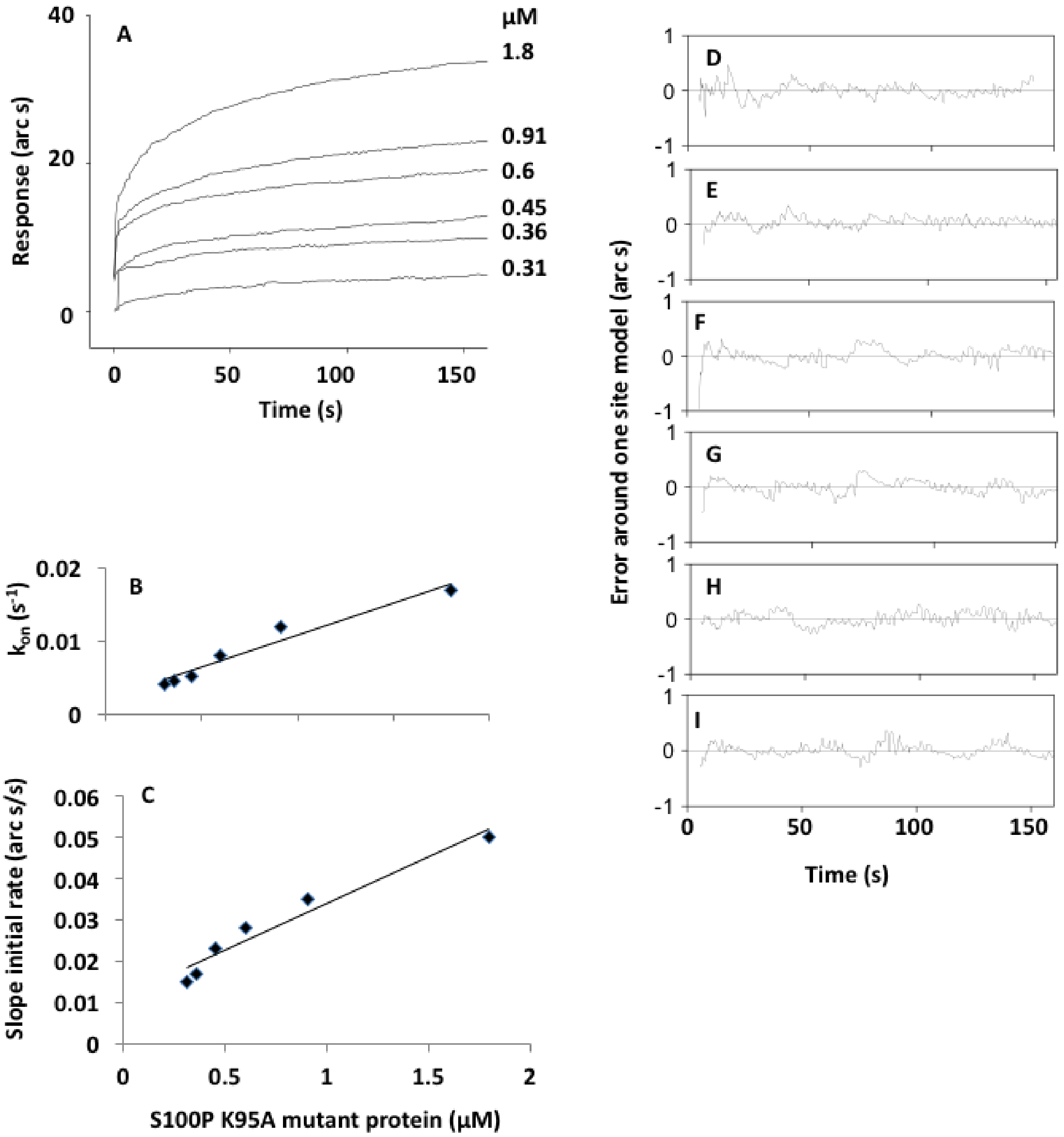

ΔK95 mutant S100P

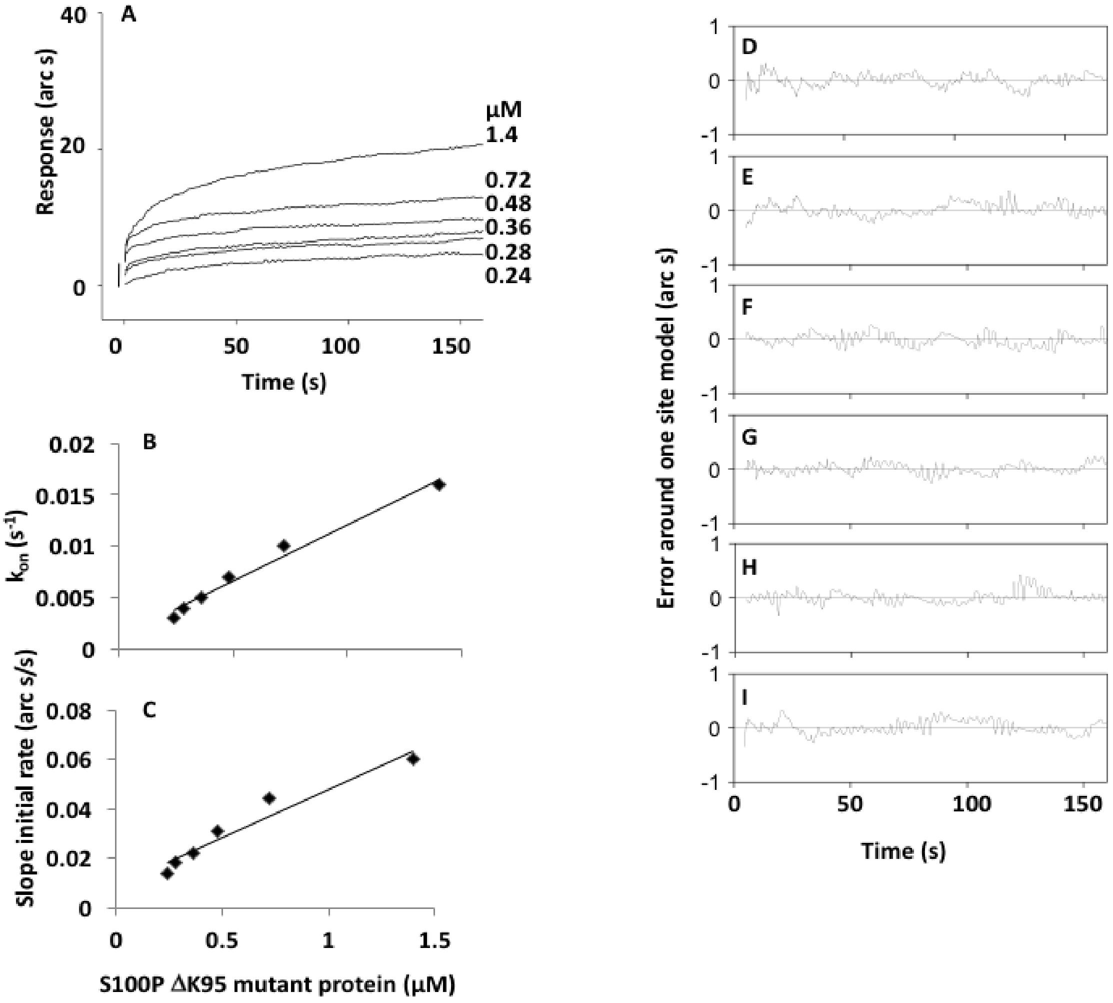

Figure S1. Kinetics of binding of mutant S100P proteins to immobilised recombinant *C*-terminal region of non-muscle myosin heavy chain, isoform A (rNMMIIA). Binding kinetics of wild type S100P, K95A mutant S100P and ΔK95 mutant S100P recombinant proteins to an immobilised fragment of recombinant (r) non-muscle myosin IIA (NMMIIA) were measured in arc sec using a plasmon resonance biosensor, as described in Materials and Methods, in the presence of 500 μM calcium ions. Each S100P protein (5 μL) at different concentrations was added to a rNMMIIA-derivatised cuvette containing 29 μL of phosphate-buffered saline to yield the final nM protein concentrations indicated for each curve. Panels **A**, association reactions (180 sec; at least 90% saturation of one-site binding curve achieved). The concentration of S100P protein increased with the observed level of binding. Data were collected three times per second. Panels **B**, linear relationship observed between on-rate constant, k_{on} (one-site model) and S100P concentrations. Panels **C**, linear relationship observed between slope of initial rate of association and S100P protein concentrations. Panels **D-I**, distributions of the data points (jagged line) around a one-site binding model (horizontal line, 0 arc s) for each concentration of protein in Panels **A** (Panels **D**, highest, Panels **I** lowest concentration). Data covered at least 90% of the curve described by the single-site model. Instrument noise is ± 0.5 arc s where 1 arc s corresponds to 1/3600 of a degree and 600 arc s equals 1 ng protein bound/mm^2

of sensor surface. These analyses suggest that the interaction of S100P proteins with rNMMIIA was monophasic and not limited by diffusion.

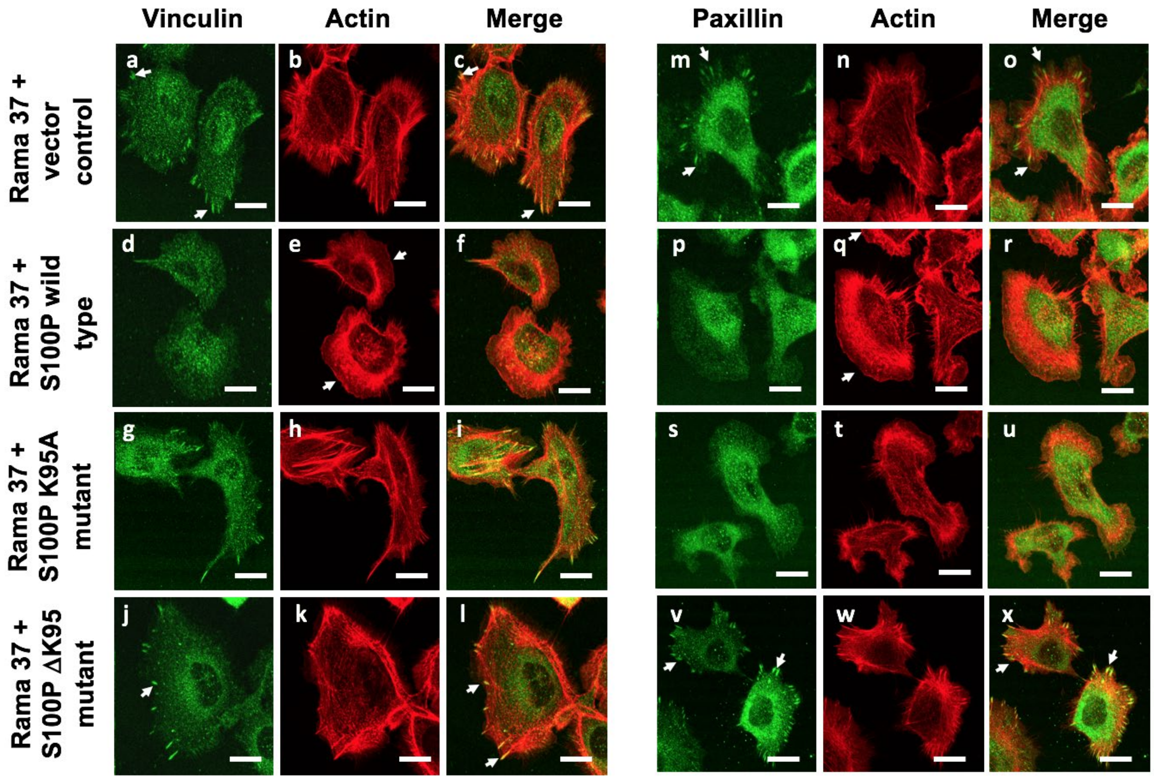

Figure S2. Immunofluorescence localisation of vinculin/paxillin focal adhesions and actin filaments in transfected cells. Rama 37 cells permanently transfected with empty expression vector (Rama 37 + vector control; a-c, m-o) or Rama 37 cells overexpressing wild-type S100P (Rama 37 + S100P wild-type; d-f, p-r), S100P K95A-mutant (Rama 37 + S100P K95A-mutant; g-i, s-u) or S100P ΔK95-mutant (Rama 37 + S100P ΔK95-mutant; j-l, v-x) were grown on fibronectin-coated coverslips for 48 hours prior to fixation, permeabilisation and staining green for vinculin (a, d, g, j) or paxillin (m, p, s, v) and red for actin (b, e, h, k, n, q, t, w). The cells were then mounted and viewed using a Zeiss LSM510 confocal laser scanning microscope. Merged images (c, f, i, l, o, r, u, x) are shown with overlap in yellow.

(**a**)

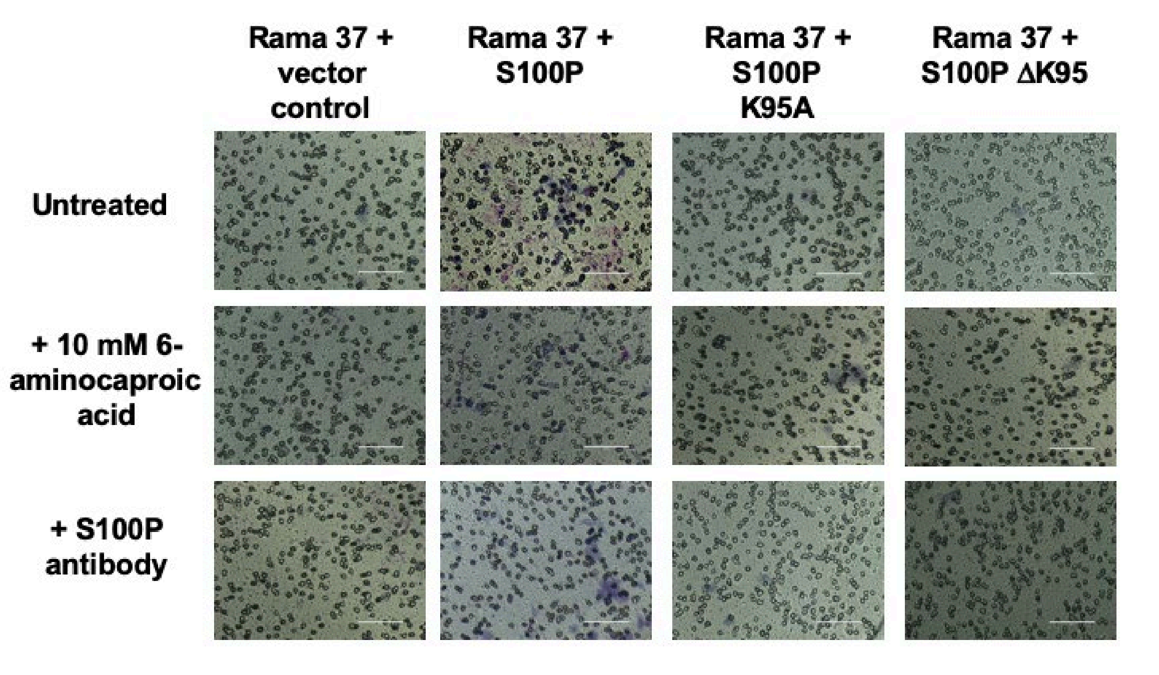

(**b**)

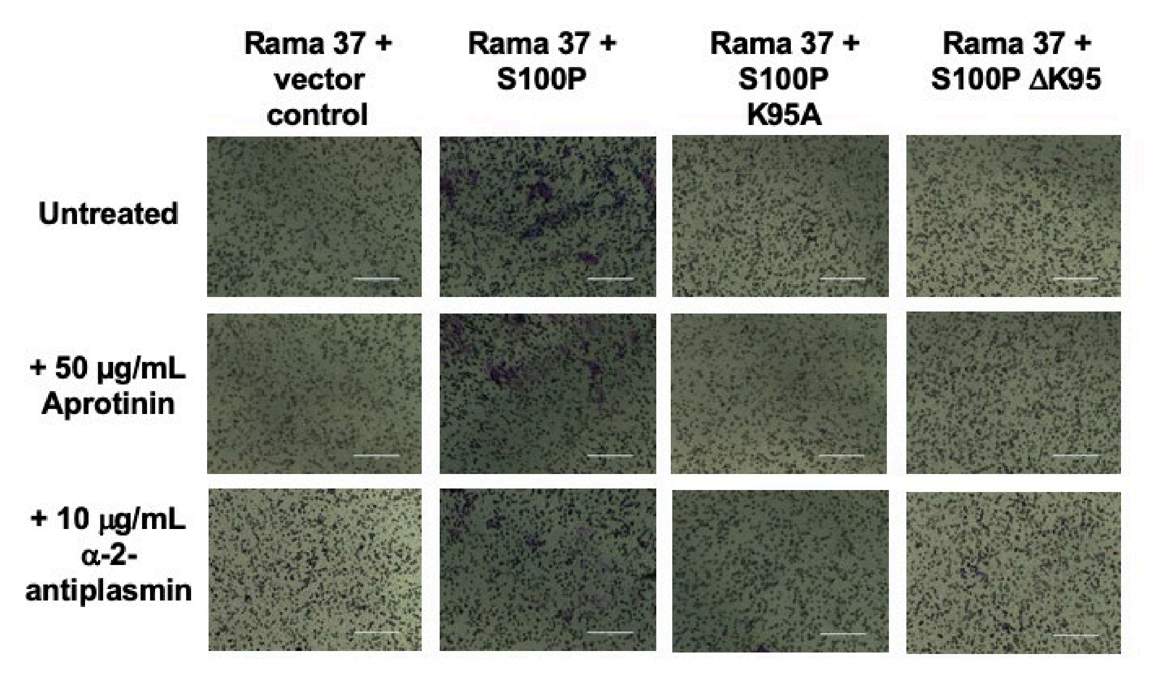

Figure S3. Representative images for Tranwell migration assays for Figure 2. Cell migration assays, using 6.5 mm diameter Transwell permeable devices with 8.0 μm pore size polycarbonate membranes, were carried out, as described previously[1], using a 0.5-10% (v/v) gradient of foetal calf serum and counting 5 random fields. (**a**) Representative images for Figure 2a. (b) Representative images for Figure 2c. White bars in all panels = 100 μm.[1] Tabrizi, M.E.A., Lancaster, T.L., Ismail, T.M., Georgiadou, A., Ganguly, A., Mistry, J.J. *et al.* S100P enhances the motility and invasion of human trophoblast cell lines. *Sci. Rep.* **8**, 11488 (2018).

Representative images of scratch migration data for Figure 2b

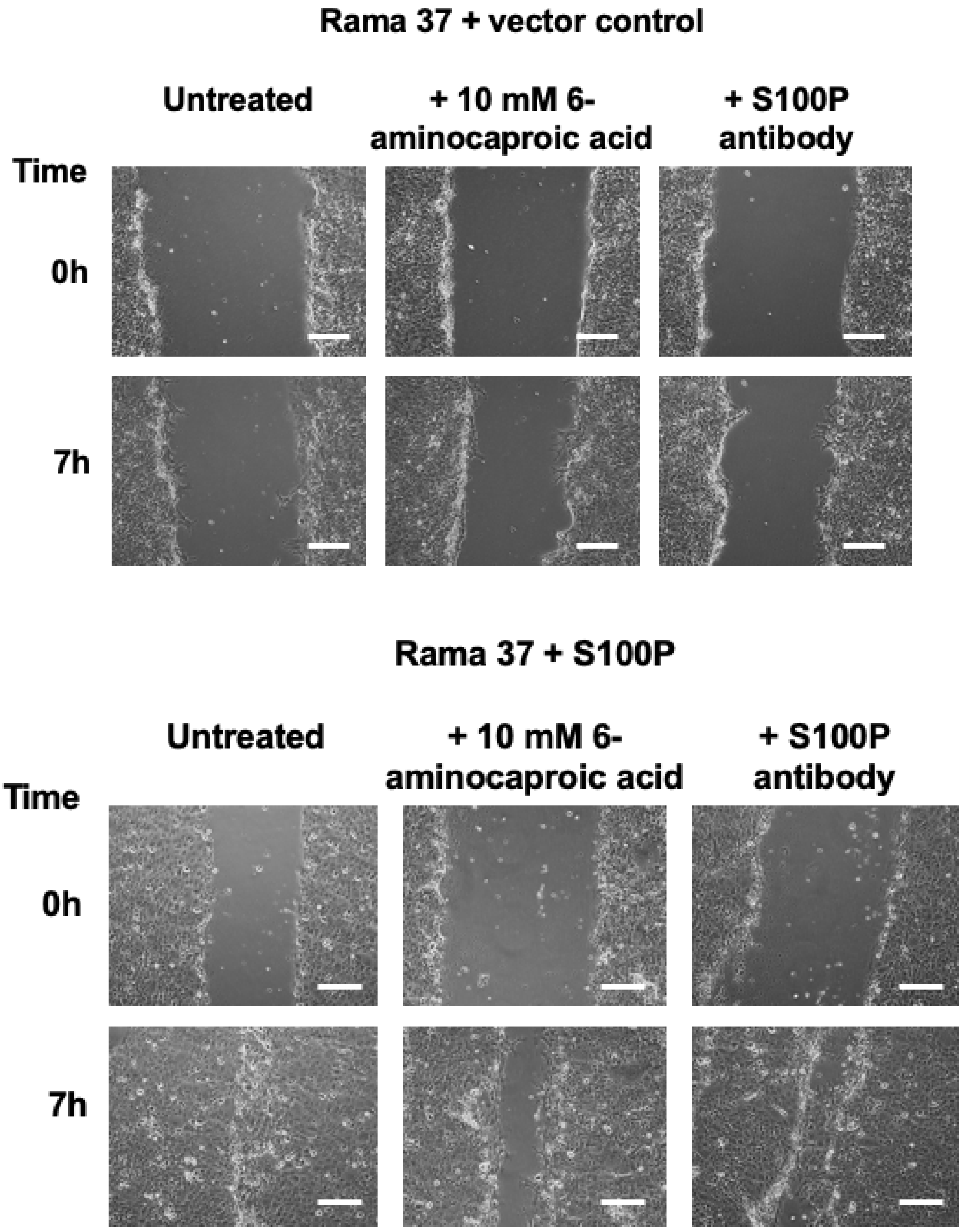

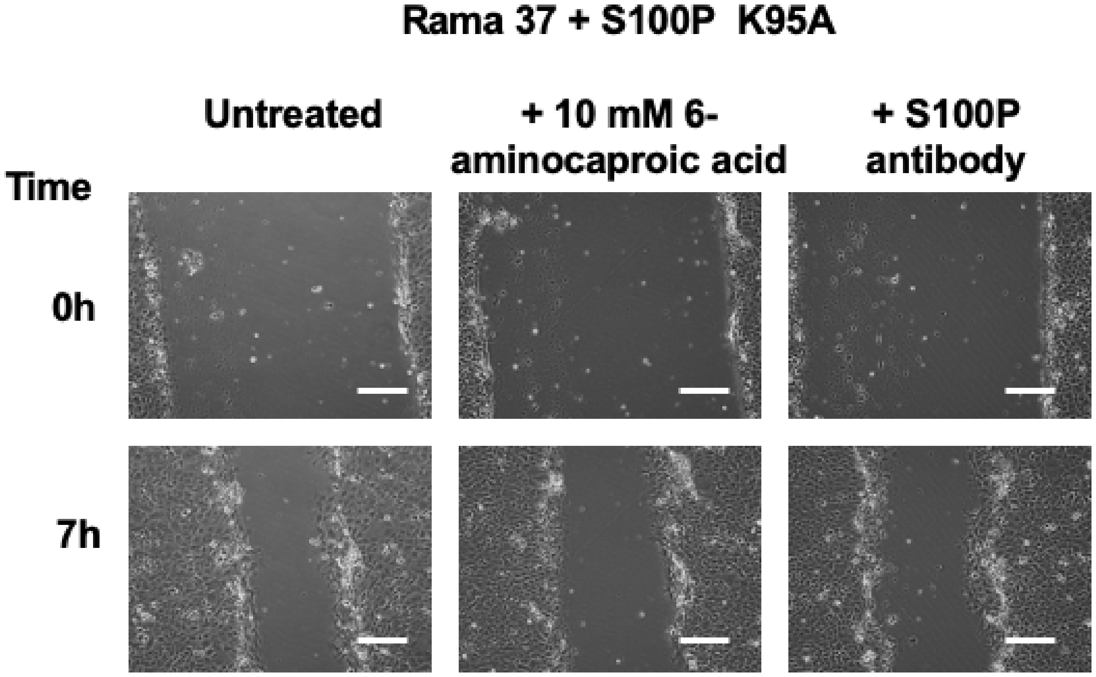

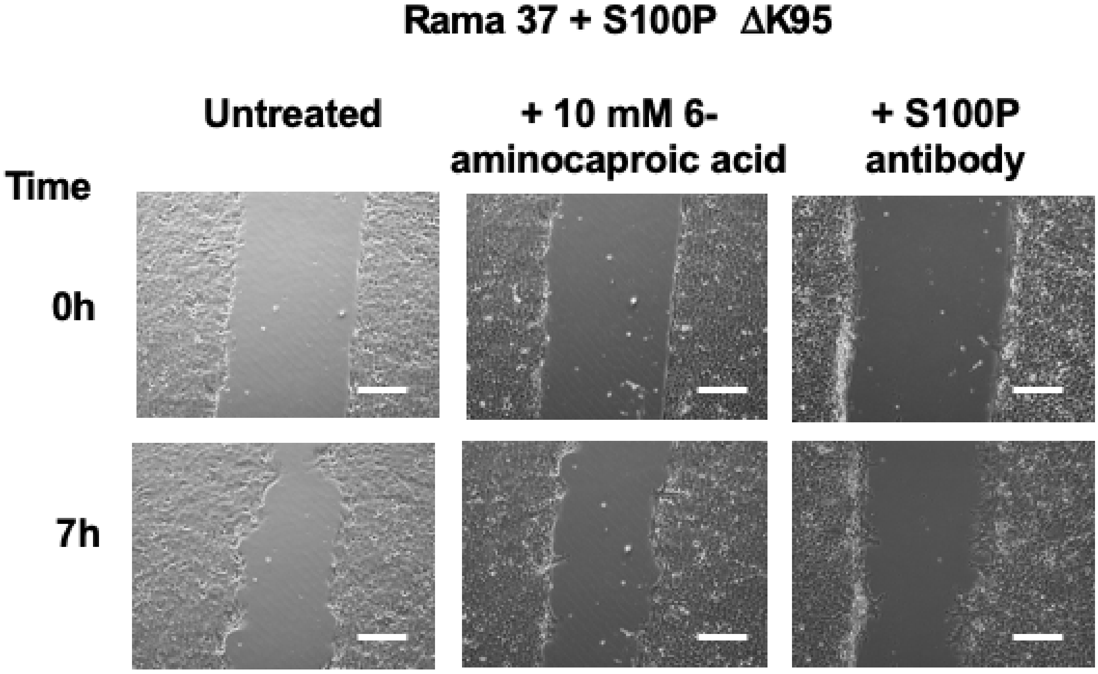

Representative images of scratch migration data for Figure 2d

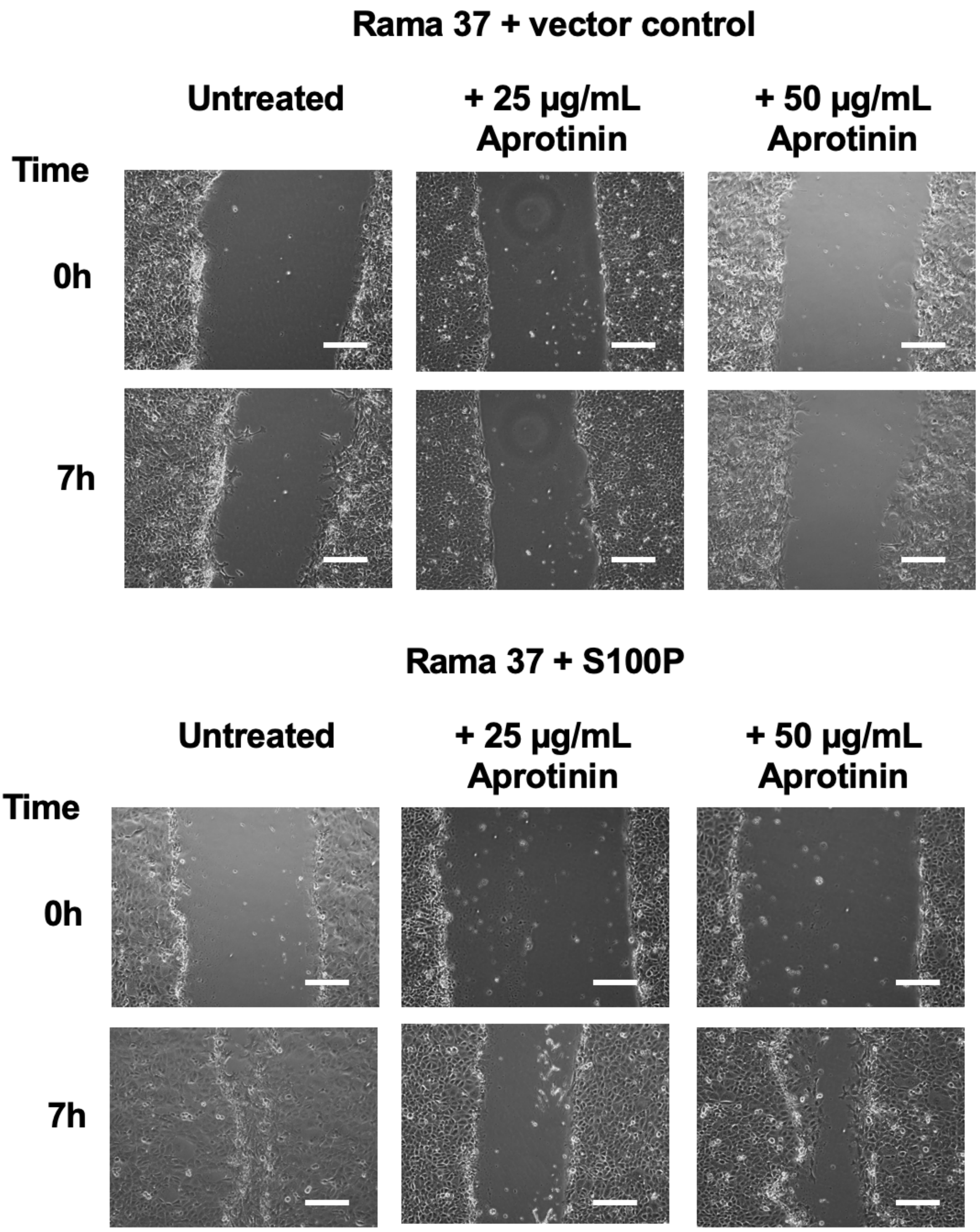
Rama 37 + vector control
Untreated
+ 25 µg/mL Aprotinin
+ 50 µg/mL Aprotinin
Time
0h
7h
Rama 37 + S100P
Untreated
+ 25 µg/mL Aprotinin
+ 50 µg/mL Aprotinin
Time
0h
7h

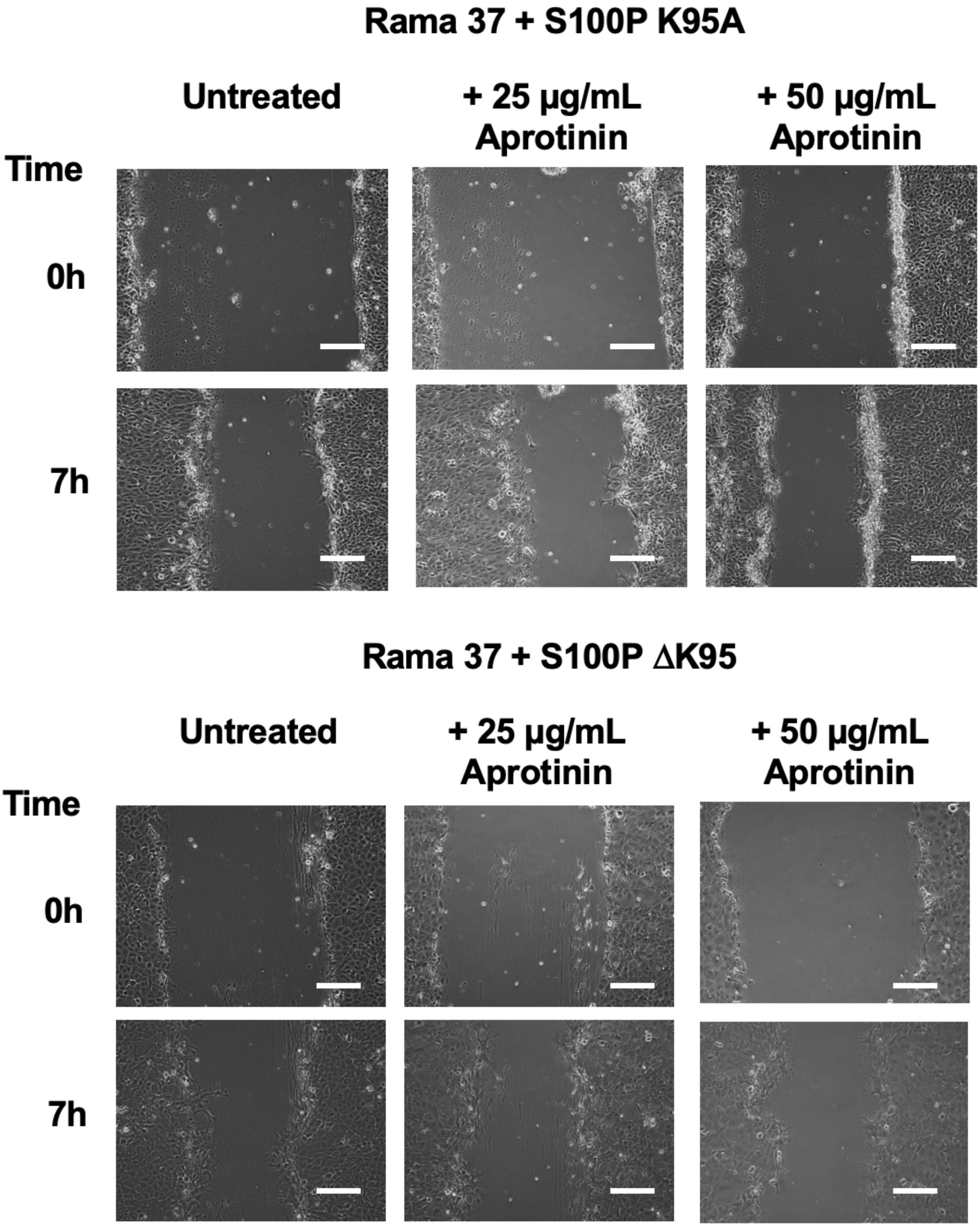

Representative images of scratch migration data for Figure 2e

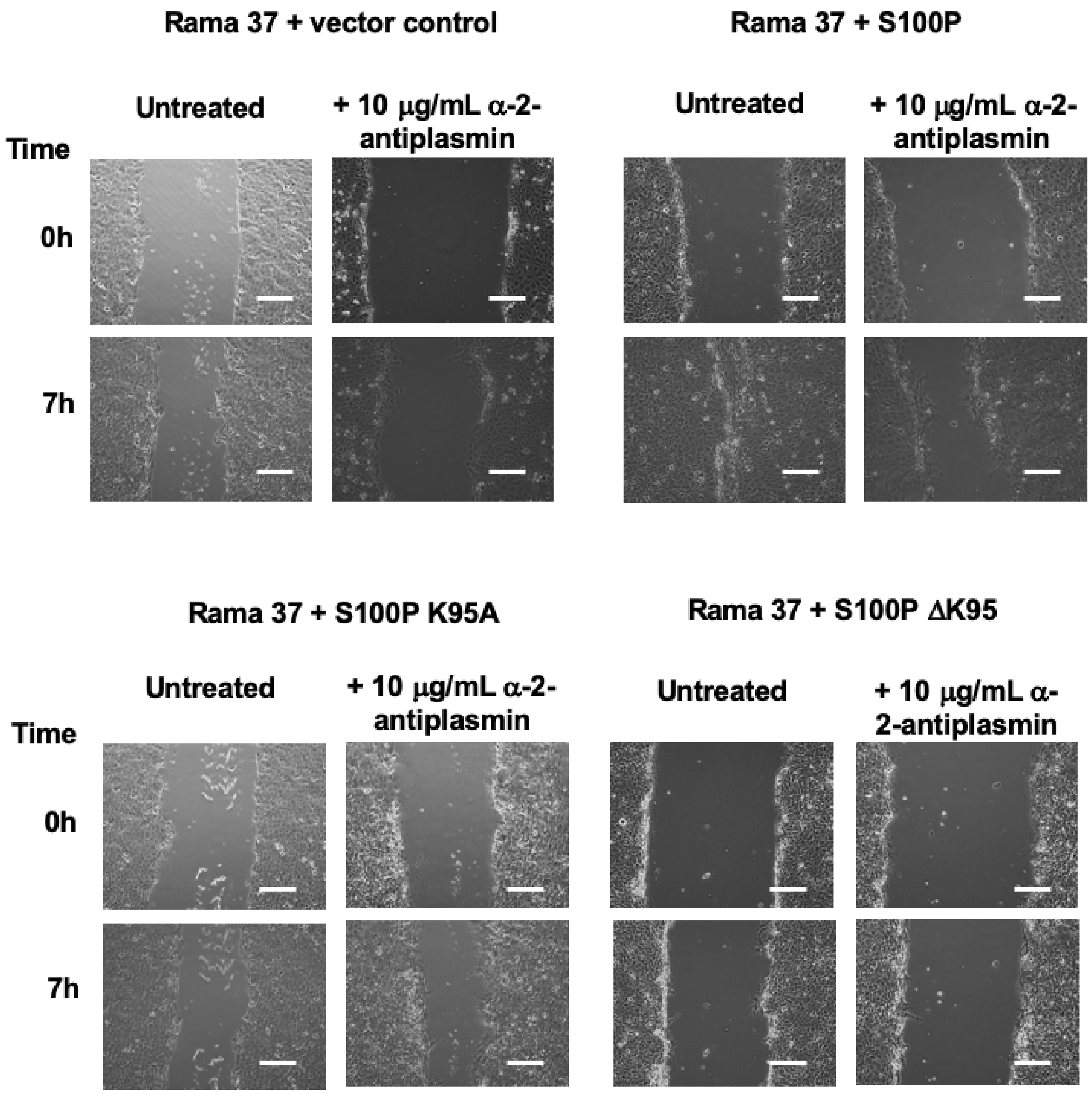

Figure S4. Representative images of scratch migration data for Figure 2. Scratch wound assays were carried out as described previously[1] using a Cell-IQ incubator (Chip-Man Technologies, Tampere, Finland). Preselected fields of the wound were imaged on a continuous loop until closure of the wound. Representative images of the scratches from the over 2,000 collected are shown for times 0 and 7 h. White bars in all panels = 200 μm. [1] Goh, C., Sin, T., Hersch, N., Rudland, P., Barraclough, R., Hoffmann, B. et al. S100A4 downregulates filopodia formation through increased dynamic instability. Cell Adhes. Migr. 5, 439-447 (2011).

Table S1. Kinetics of binding of wild-type and mutant recombinant S100P proteins to immobilised recombinant C-terminal fragment of non-muscle myosin II heavy chain.

**Protein**[a]	$k_{ass.}$ ± SE ($M^{-1}s^{-1}$)[b]	**Corr. coeff** [c]	$k_{diss.}$ ± SE (s^{-1}) [d]	K_d ± SE (kinetic) (nM) [e]	K_d ± SE (equilibrium) (nM) [f]
Wild-type S100P	9,900±3,300	0.93	0.0046±0.0008	466±160	378±28

K95A-mutant S100P	8,330±2,600	0.92	0.035±0.0001	4,100±1,300	2,300±2,300
ΔK95 mutant S100P	9,000±3,000	0.93	0.06±0.01	6,000±2,200	2,700±540

[a] K95A-mutant S100P and ΔK95-mutant S100P refer to S100P proteins with C-terminal lysine substituted with alanine or deleted, respectively. [b] k_{ass} and S.E. from three experiments are derived as described previously[1].

[c] Correlation coefficient of the linear regression through k_{on} values used for obtaining k_{ass}. [d] k_{diss} of the mean ± SE of three values at high concentrations of S100P. No evidence was found for a two-site model of dissociation, suggesting that the S100P binding sites on NMMIIA were homogeneous. [e] K_d kinetic was calculated from the ratio of k_{diss}/k_{ass} ± SE. The SE is the combined SE of the two kinetic parameters. [f] K_d equilibrium ± SE was calculated from the extent of binding near equilibrium, as described previously[1].

1. Chen H, Fernig DG, Rudland PS, Sparks A, Wilkinson MC, Barraclough R. Binding to intracellular targets of the metastasis-inducing protein, S100A4 (p9Ka). *Biochem Biophys Res Commun* 2001; **286:** 1212-1217.

Table S2. Quantitation in S100P expressing cells of the myosin A fibre pattern that predominates in S100P-negative Rama 37 cells.

Panel A

Cell clone	Percentage of cells/field showing non-muscle myosin IIA-staining fibre type found predominantly in S100P negative control cells. 7 fields examined for each cell clone.	Mean percentage value ± SD	P value in comparison with S100P- negative Rama 37 + vector control
S100P-negative Rama 37 + vector control	100, 66.7, 50, 100, 100, 0, 100	73.8 ± 38.3	-
Rama 37 + wild type S100P	0, 0, 0, 0, 0, 0, 25	3.6 ± 9.4	P<0.0001
Rama 37 + S100P K95A	100, 100, 100, 100, 50, 100, 75	89.3 ± 19.7	P = 0.506
Rama 37 + S100P ΔK95	33.3, 100, 66.7, 50, 60, 66.7, 75	64.5 ± 20.7	P = 0.818

Panel B

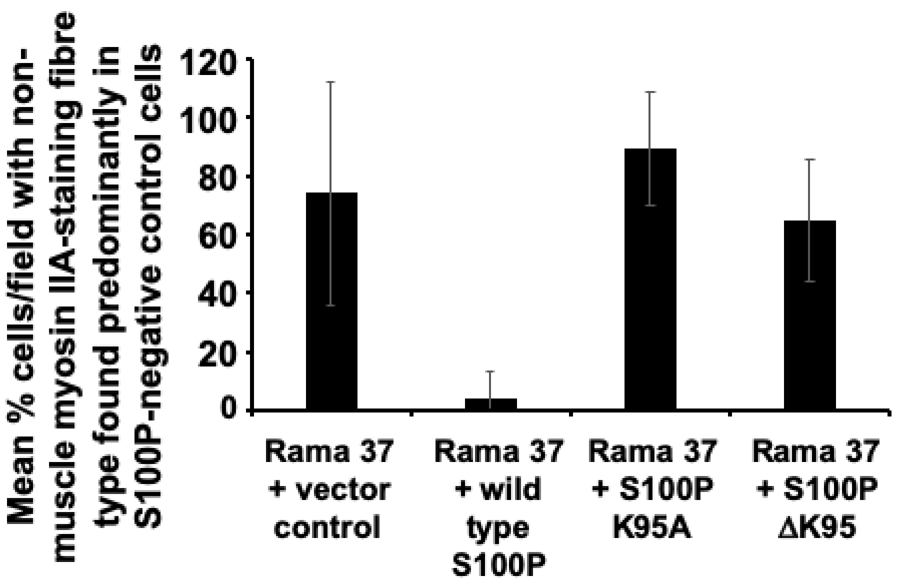

Rama 37 cells expressing wild-type S100P, S100P mutant proteins K95A or ΔK95 or S100P-negative control cells were fluorescently stained as described in Materials and Methods with antibodies to non-muscle myosin IIA (Covance, now Biolegend, Dedham, USA, PRB-440P, 0.5 μg/mL). Examples of staining are shown in Figure 1 of the main manuscript. Cells in 7 microscopic fields for each cell line were scored for the proportion of cells with non-muscle myosin IIA fibre type that predominated in the S100P-negative cells as described previously[1]. There was no significant difference in myosin arrangement between cells expressing K95A or ΔK95 mutant S100P proteins and the S100P-negative control cells using ANOVA with post-hoc Dunnett multiple comparisons with a control, however there was a highly significant 20.5-fold reduction in cells expressing wild-type S100P (panel A). The mean results from panel A are plotted as mean with error bars ± SD (panel B) for clarity.

[1]. Du, M., Wang, G., Ismail, T.M., Gross, S., Fernig, D.G., Barraclough, R. *et al.* S100P dissociates myosin IIA filaments and focal adhesion sites to reduce cell adhesion and enhance cell migration. *J. Biol. Chem.* **287**, 15330-15344 (2012).

Table S3. Quantitation in S100P-expressing cells of the myosin B fibre pattern that predominates in S100P-negative Rama 37 cells.

Panel A

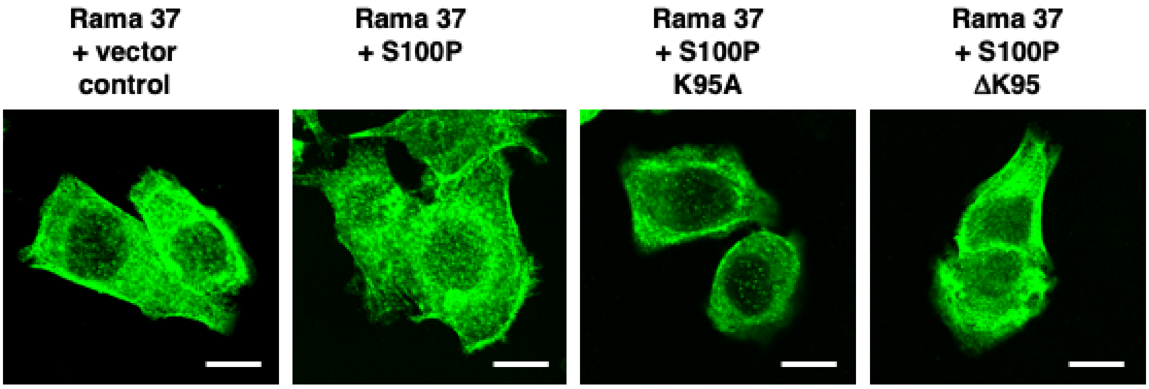

Panel B

Cell clone	Percentage of cells/field showing non-muscle myosin IIB-staining fibre type found predominantly in S100P negative control cells. 6 fields examined for each cell clone.	Mean percentage value ± SD	P value in comparison with S100P- negative Rama 37 + vector control
S100P-negative Rama 37 + vector control	100, 0, 100, 100, 100, 100	83.3 ± 40.8	-
Rama 37 + wild type S100P	66.7, 100, 100, 66.7, 100, 100	88.9 ± 17.2	P = 0.973
Rama 37 + S100P K95A	100, 100, 100, 100, 50, 100	91.7 ± 20.4	P = 0.919
Rama 37 + S100P ΔK95	33.3, 100, 66.7, 100, 100, 100	83.3 ± 27.9	P>0.9999

Panel C

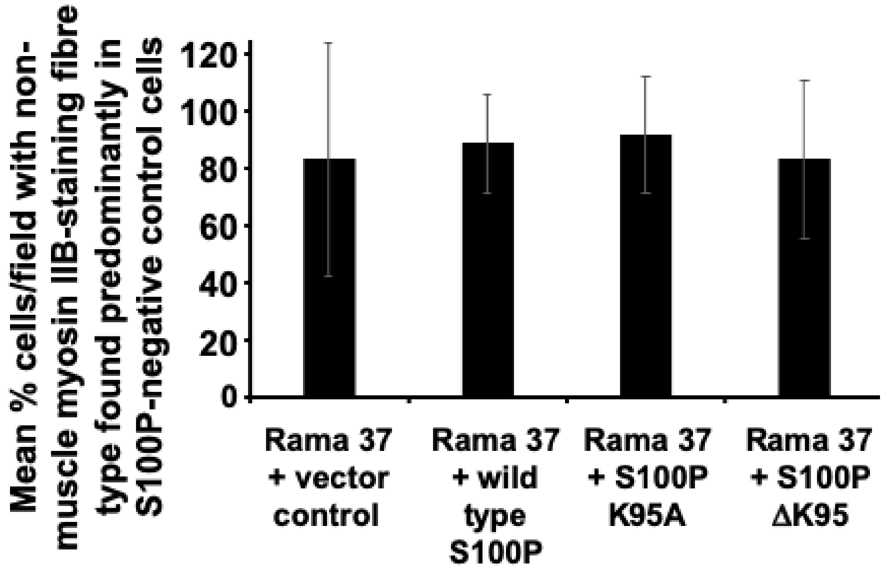

Rama 37 cells expressing wild-type S100P, S100P mutant proteins K95A or ΔK95 or S100P-negative control cells were fluorescently stained as described in Materials and Methods with antibodies to non-muscle myosin IIB (Covance, now Biolegend, Dedham, USA, PRB-445P, 1μg/mL). Examples of staining are shown in panel A, bar = 10 μm. Cells in 6 microscopic fields for each cell line were scored for the proportion of cells with non-muscle myosin IIB fibre type that predominated in the S100P-negative cells as described previously[1]. There was no significant difference in myosin arrangement between cells expressing S100P/mutant S100P proteins and the S100P-negative control cells using ANOVA with post-hoc Dunnett multiple comparisons with a control (panel B). The mean results from panel B are plotted as mean with error bars ± SD (panel C) for clarity.

[1]. Du, M., Wang, G., Ismail, T.M., Gross, S., Fernig, D.G., Barraclough, R. *et al.* S100P dissociates myosin IIA filaments and focal adhesion sites to reduce cell adhesion and enhance cell migration. *J. Biol. Chem.* **287**, 15330-15344 (2012).

Table S4. Effect of 6-aminocaproic acid and S100P antibody on the number of vinculin- and paxillin-stained focal adhesions in S100P-negative and wild-type S100P-expressing cells.

	Focal vinculin[a]			Focal paxillin[a]		
**Cell clone and treatment**	**No of cells counted**	**Mean focal adhesions/cell ± SD[b]**	**Mean focal adhesions as % of untreated vector control**	**No of cells counted**	**Mean focal adhesions/cell ± SD[b]**	**Mean focal adhesions as % of untreated vector control**
Vector control untreated	22	20.9 ± 10.3	100	12	29.3 ± 16	100
+ ACA	18	20.4 ± 11.4*	97.7	11	25.3 ± 15.9*	86.2

+S100P antibody	15	18.3 ± 12.4**	87.9	15	27.5 ± 12.9**	93.6
S100P expressing cells	15	4.6 ± 3.2§	22.0	16	8.7 ± 5.4§	29.6
+ ACA	14	6.1 ± 5.7¶	29.1	18	7.8 ± 6.3¶	26.5
+ S100P antibody	9	3.8 ± 3.4¶¶	18.1	21	10.4 ± 8.9¶¶	35.4

[a] Cloned S100P-negative vector control cells or cloned S100P-expressing Rama 37 cell grown on fibronectin-coated glass coverslips were treated with 10 mM 6-aminocaproic acid (ACA) or 1,000-fold dilution of S100P antibody or left untreated and stained for vinculin or paxillin as described in Materials and Methods and Supplementary Methods M1. Vinculin or paxillin-stained focal adhesions were counted in 9-22 cells and the mean and standard deviation of the total number of stained focal adhesions per cells were calculated.
[b] Significances of differences were calculated using Mann-Whitney *U*-test. * Number of vinculin or paxillin focal adhesions per cell not significantly different from untreated vector control (p = 0.877 and 0.618, respectively). ** Number of vinculin or paxillin focal adhesions per cell not significantly different from untreated vector control (p = 0.476 and 0.54, respectively). § Number of vinculin or paxillin focal adhesions per cell in S100P-expressing cells significantly fewer than untreated vector control (p <0.0001 and P = 0.0017, respectively). ¶ Number of vinculin or paxillin focal adhesions per cell not significantly different from untreated S100P expressing cells (p = 0.614 and 0.488, respectively). ¶¶Number of vinculin or paxillin focal adhesions per cell not significantly different from untreated S100P-expressing cells (p = 0.369 and 0.972, respectively).

Table S5. Effect of aprotinin and α-2-antiplasmin on the number of paxillin-staining focal adhesions in S100P-negative and wild-type S100P-expressing cells

**Cell clone and treatment**	**Focal paxillin[a]**		
	**No. of cells counted**	**Mean focal adhesions/cell ± SD[b]**	**Mean focal adhesions as % of vector control**
Vector control untreated	41	62.9 ± 19.5	100
Vector control + aprotinin	42	60.8 ± 19.5*	96.7
Vector control + α-2-antiplasmin	58	47.9 ± 17.8§	76.2
S100P expressing cells untreated	58	9.5 ± 9.7§§	15.1
S100P-expressing cells + aprotinin	63	10.1 ± 16.9¶	16.1
S100P-expressing cells + α-2-antiplasmin	65	8.8 ± 10.5¶¶	14.0

[a] Cloned cell lines grown on fibronectin-coated glass coverslips were treated with 50 μg/mL aprotinin or 10 μg/mL α-2-antiplasmin and stained for paxillin as described in Materials and methods. Paxillin-stained focal adhesions were counted in 40-65 cells and the mean and standard deviation of the total number per cells was calculated. [b] Significance of differences were calculated using Mann-Whitney *U*-test. * Number of paxillin focal adhesions per cell not significantly different from untreated vector control (p = 0.5). § Number of paxillin

focal adhesions per cell significantly fewer than untreated vector control (p = 0.0003). §§ Number of paxillin focal adhesions per cell in untreated wild type S100P-expressing cells significantly fewer than in untreated S100P-negative, vector control cells (p <0.0001). ¶ Number of paxillin focal adhesions per cell not significantly different from untreated S100P expressing cells (p = 0.19). ¶¶Number of paxillin focal adhesions per cell not significantly different from untreated S100P-expressing cells (p = 0.4).

Supplementary Methods S1

Details of site directed mutagenesis of wild type S100P cDNA

A cDNA encoding the wild-type S100P protein in pET15b vector was subjected to site-directed mutagenesis using a Quikchange™ site-directed mutagenesis kit (Stratagene, La Jolla, CA). A K95A mutant in which the C-terminal lysine was replaced by alanine was created by mutating codon 95, which encodes the C-terminal lysine, from AAA to GCA using the following pair of complementary oligonucleotide primers: forward 5′ GAGAAGGCAGGACTCGCATGATGCCCTGGAGATGTC 3′, reverse 5′ GACATCTCCAGGGCATCATGCGAGTCCTGCCTTCTC 3′. A ΔK95-mutant was generated by converting codon 95 from AAA to terminator TAA using the following primers: forward 5′ GAGAAGGCAGGACTCTAATGATGCCCTGGAGATGTC 3′,
reverse 5′ GACATCTCCAGGGCATCATTAGAGTCCTGCCTTCTC 3′. Recombinant proteins were produced, as described previously[1,2]. The C-terminal amino acids of recombinant wild-type and mutant S100P proteins were confirmed by mass spectroscopic analysis of recombinant proteins, as follows. Recombinant S100P proteins were digested with trypsin in 10 mM ammonium bicarbonate and the resulting digests were analysed by electrospray MS. This produced separate C-terminal peptides of masses 388.3 (wild type, AGLK theoretical), 260.1 (ΔK95-mutant, AGL theoretical), 331.2 (K95A-mutant, AGLA theoretical), which were confirmed by sequencing using MS-MS.

Details of immunofluorescence staining of cultured cells

Rama 37 cells (15,000 cells) expressing wild-type S100P, K95A S100P or ΔK95 S100P, or cells transfected with empty vector were plated onto fibronectin-coated (2.5 μg/cm^2) glass coverslips in 24-well plates. Cells were fixed, permeabilised and blocked, as described previously[3], before being incubated with primary antibodies against non-muscle myosin IIA (Covance, now Biolegend, Dedham, USA, PRB-440P), non-muscle myosin IIB (Covance, now Biolegend, Dedham, USA, PRB-445P), S100P (BD Transduction Laboratories, Oxford, UK or R&D systems, Abingdon, U.K.), vinculin (Sigma, St Louis, USA), paxillin (Invitrogen, Paisley, UK) or eEF1A (clone CBP-KK1, mouse, Millipore, UK), appropriately-diluted in blocking solution (1% (v/v) goat serum in cytoskeleton buffer (150 mM NaCl, 5 mM $MgCl_2$, 5 mM EGTA, 5 mM glucose, 10 mM 2-(N-morpholino) ethanesulfonic acid, pH 6.1)) for 45 min at 37 °C. After three washes with blocking solution, cells were incubated with the appropriate secondary antibodies, anti-rabbit or anti-mouse antibodies conjugated with fluoroscein isothiocyanate (Dako, Ely, UK) or Cy3 dye (Stratech Scientific, Norfolk, UK), respectively, in blocking solution for 45 min at 37 °C. For actin staining, 0.6 μM rhodamine phalloidin (Invitrogen, Paisley, UK) was added with secondary antibodies. After washing with blocking solution, coverslips were rinsed once with water and mounted in Vectashield mounting medium (Vector, Peterborough, UK), before being viewed using a Zeiss LSM510 confocal laser

scanning microscope. Quantitation of the immunofluorescence patterns of non-muscle myosin IIA and IIB was carried out, as described previously[4]. For quantification of focal adhesions their numbers per cell were counted in about 50 randomly-selected cells from 3 independent experiments.

1 Ismail T, Fernig D, Rudland P, Terry C, Wang G, Barraclough R. The basic C-terminal amino acids of calcium-binding protein S100A4 promote metastasis *Carcinogenesis* 2008; **29**: 2259-2266.

2 Ismail TM, Zhang S, Fernig DG, Gross S, Martin-Fernandez ML, See V *et al*. Self-association of calcium-binding protein S100A4 and metastasis. *J Biol Chem* 2010; **285**: 914-922.

3 Tabrizi MEA, Lancaster TL, Ismail TM, Georgiadou A, Ganguly A, Mistry JJ *et al*. S100P enhances the motility and invasion of human trophoblast cell lines. *Sci Rep* 2018; **8**: 11488.

4 Du, M., Wang, G., Ismail, T.M., Gross, S., Fernig, D.G., Barraclough, R. *et al.* S100P dissociates myosin IIA filaments and focal adhesion sites to reduce cell adhesion and enhance cell migration. *J Biol Chem* 2012; **287**, 15330-15344.
